# Targeting TGF-β–driven fibrotic, epigenetic remodeling and Bax/Bcl-2 through polyphenol–exercise synergy: molecular implications for biomechanical impairment and rehabilitation in cancer

**DOI:** 10.3389/fonc.2026.1813474

**Published:** 2026-04-29

**Authors:** Lin Liu

**Affiliations:** School of General Education, Zhoukou Vocational College of Arts and Sciences, Zhoukou, Henan, China

**Keywords:** activity analysis, injury prevention, performance optimization, polyphenols, rehabilitation

## Abstract

Optimizing human activity through biomechanical efficiency is central not only to enhancing physical performance but also to restoring functional capacity in disease states. In cancer patients, tumor progression and treatment-related toxicities induce profound molecular and epigenetic alterations within the tumor–muscle–matrix axis, leading to fibrosis, muscle wasting, neuromuscular dysfunction, and impaired activity biomechanics. While exercise training remains a cornerstone intervention for improving activity quality and physical resilience, emerging evidence suggests that targeted nutritional strategies, particularly polyphenol supplementation, may modulate key oncogenic and fibrotic signaling pathways. Polyphenols, a diverse class of plant-derived bioactive compounds, exert potent antioxidant, anti-inflammatory, and epigenetic regulatory effects that influence TGF-β/SMAD signaling, microRNA expression, extracellular matrix remodeling, and mitochondrial function in cancer and skeletal muscle tissues. This review examines the molecular and epigenetic convergence of polyphenols and exercise in reprogramming tumor–muscle–matrix crosstalk, and discusses how these mechanisms translate into biomechanical dysfunction, injury susceptibility, and rehabilitation outcomes in cancer patients. By integrating data from preclinical oncology models, clinical studies in cancer populations, and mechanobiological research, we explore how combined nutritional–mechanical interventions may attenuate cancer-associated fibrosis, modulate inflammatory and epigenetic signatures, preserve muscle integrity, and improve functional activity capacity. We further propose that selective modulation of fibrotic and epigenetic pathways may reduce biomechanical impairment, enhance rehabilitation responsiveness, and mitigate injury risk during cancer survivorship. The concept of molecular–mechanobiological synergy provides a translational framework linking tumor biology to functional activity outcomes. Future interdisciplinary research should prioritize longitudinal, multimodal studies integrating epigenetic biomarkers, tumor-derived signaling mediators, and advanced biomechanical profiling in oncology settings to validate and personalize combined exercise–polyphenol strategies.

## 1 Introduction

Cancer and its therapeutic interventions precipitate significant structural, molecular, and functional modifications that transcend the confines of the primary neoplasm, thereby contributing to enduring biomechanical dysfunction and reduced rehabilitative potential in survivors. Among the most pivotal yet insufficiently integrated catalysts of these modifications are transforming growth factor-β (TGF-β)–mediated fibrotic remodeling, ongoing epigenetic reprogramming, and the dysregulation of apoptosis as a result of the imbalance between Bax and Bcl-2 proteins (1, 2).

TGF-β is extensively acknowledged as a pleiotropic cytokine that regulates extracellular matrix (ECM) synthesis, fibroblast activation, epithelial–mesenchymal transition (EMT), and immunological modulation (3, 4). In a multitude of malignancies, as well as subsequent to radiotherapy or chemotherapy, prolonged TGF-β signaling fosters the differentiation of myofibroblasts and the excessive deposition of collagen, culminating in tissue rigidity, contractures, and functional deterioration (5, 6). These fibrotic mechanisms not only facilitate tumor advancement but also contribute to compromised mobility, diminished muscle elasticity, and impaired organ compliance, thereby directly influencing rehabilitation outcomes. Beyond its established Smad-dependent signaling pathways, TGF-β engages with a multitude of non-Smad signaling cascades and modulates the transcriptional landscape via epigenetic mechanisms. Abnormalities in epigenetic processes have been observed across a variety of cancer types, thereby influencing gene expression programs that promote pro-fibrotic and pro-survival phenotypes (7, 8). Nevertheless, despite the extensive review of the epigenetic aspects of cancer biology, the direct connection to biomechanical impairment and the responsiveness to rehabilitation remains inadequately investigated.

Apoptotic regulation represents a pivotal intersection between the domains of cancer biology and tissue regeneration. The equilibrium established between pro-apoptotic proteins, exemplified by Bax, and anti-apoptotic counterparts such as Bcl-2, critically influences cellular fate in response to stressors (9). In oncological contexts, the elevated expression of Bcl-2 coupled with the downregulation of Bax fosters the survival of tumor cells and contributes to resistance against therapeutic interventions; conversely, in non-malignant tissues subjected to cytotoxic therapies, an overabundance of apoptosis may aggravate muscle wasting, vascular injury, and organ dysfunction (10).

The signaling pathways of TGF-β have been demonstrated to exert significant influence over apoptotic mechanisms, either facilitating or restraining apoptosis contingent upon the cellular milieu and the stage of oncogenesis (3). Notwithstanding the comprehensive examination of Bax/Bcl-2 interactions in tumor progression, their contribution to the processes of tissue remodeling and functional deterioration following treatment especially within the musculoskeletal and cardiopulmonary systems, has been subject to comparatively sparse integrative scrutiny.

Exercise has increasingly been recognized as a fundamental component of supportive oncology care, yielding significant advancements in the domains of fatigue reduction, cardiorespiratory fitness enhancement, muscle strength augmentation, and overall quality of life improvement (11, 12). From a mechanistic perspective, exercise has been shown to modulate the levels of inflammatory cytokines, regulating epigenetic modifications, enhance mitochondrial functionality, and potentially diminish TGF-β expression in specific contexts associated with fibrosis (13–15).

In a concurrent manner, dietary polyphenolic compounds including resveratrol, curcumin, epigallocatechin gallate (EGCG), and quercetin, have exhibited properties that are anti-inflammatory, antioxidant, anti-fibrotic, and capable of modulating epigenetic mechanisms (16, 17). Various polyphenolic substances inhibit TGF-β signaling pathways, attenuate the activation of myofibroblasts, and modulate the activity of Bcl-2 family proteins, thereby exerting effects on both fibrotic processes and apoptotic pathways (18). Furthermore, polyphenols may serve as epigenetic regulators through their influence on DNA methyltransferases and histone deacetylases, thereby holding the potential to reverse dysregulated transcriptional programs (19–21).

Crucially, the potential synergistic effects between structured physical exercise and polyphenol supplementation in addressing common molecular pathways have not been thoroughly synthesized within the framework of biomechanical dysfunction associated with cancer.

Existing literature has predominantly assessed TGF-β in the context of tumor progression, polyphenols concerning cancer chemoprevention, and exercise with regard to survivorship outcomes as distinct domains. To this point, there has been no exhaustive review that has systematically integrated TGF-β–induced fibrosis, epigenetic alterations, and Bax/Bcl-2–regulated apoptosis within a cohesive framework that also encompasses the synergistic effects of polyphenols and exercise. From both a scientific and clinical standpoint, there exists a pressing necessity to consolidate molecular oncology with the principles of rehabilitation biology. By amalgamating evidence from molecular signaling, epigenetics, apoptosis, nutrition, and exercise physiology, this review aspires to offer a novel, integrative perspective that may inform future experimental research, clinical trials, and personalized rehabilitation strategies.

In this narrative review, we specifically examine the molecular and epigenetic convergence of polyphenols and exercise in reprogramming tumor–muscle–matrix crosstalk, with a particular focus on TGF-β–driven fibrotic remodeling, Bax/Bcl-2-related apoptotic balance, and associated microRNA and extracellular matrix alterations in the context of cancer.

## 2 Conceptualizing nutritional–mechanical synergy in physical activity and adaptation

Physical performance emerge from the dynamic integration of mechanical loading, metabolic availability, and biological recovery processes (22). While mechanical stress imposed by exercise is widely recognized as the primary driver of neuromuscular adaptation, the magnitude, quality, and sustainability of this adaptation are strongly influenced by the surrounding nutritional environment. In recent years, this interaction has increasingly been conceptualized not as a simple additive effect, but as a synergistic relationship in which nutritional factors modulate how tissues perceive, respond to, and recover from mechanical stimuli (23). Within this perspective, nutritional–mechanical synergy refers to the capacity of dietary inputs to shape the biological context in which exercise-induced loading is processed, thereby influencing downstream activity outcomes. Rather than acting as direct ergogenic agents, nutritional compounds modify the internal conditions governing adaptation, including substrate availability, redox balance, inflammatory signaling, and cellular resilience. These factors collectively influence the efficiency of force generation, neuromuscular coordination, and tissue repair following exercise (24, 25).

Importantly, adaptation to exercise is not dictated solely by the absolute magnitude of mechanical load, but by the organism’s ability to tolerate, recover from, and adapt to repeated stress exposures. Nutritional status plays a critical role in this process by shaping recovery kinetics and the balance between transient damage and effective repair. In this context, optimal adaptation reflects not the maximal suppression of exercise-induced stress, but the regulation of stress responses within a physiological range that supports remodeling and functional improvement (26, 27). Consequently, alterations in strength, power, coordination, or activity quality represent the cumulative expression of molecular and cellular processes shaped by both mechanical loading and nutritional modulation (28, 29). Building on this foundation, the functional relevance of nutritional–mechanical synergy becomes clearer when molecular effects are traced across biological scales toward activity-level expression.

At the cellular level, polyphenols such as curcumin, resveratrol, and quercetin modulate oxidative balance, calcium handling, and stress-responsive signaling pathways, including nuclear factor kappa-light-chain-enhancer of activated B cells (NF-κB), nuclear factor erythroid 2–related factor 2(Nrf2), and AMP-activated protein kinase (AMPK)-related networks (30, 31). These pathways are tightly linked to mitochondrial efficiency, membrane stability, and excitation–contraction coupling, all of which are fundamental determinants of force generation and fatigue resistance.

Evidence from both human and animal studies indicates that improvements in activity performance can occur in the absence of complete normalization of inflammatory or oxidative biomarkers, suggesting that functional recovery may precede biochemical resolution (32, 33).

At the level of integrated activity, relatively small shifts in neuromuscular efficiency or tissue resilience can produce disproportionately large effects on performance expression. Metrics such as jump mechanics, sprint power, or endurance capacity are highly sensitive to changes in coordination, force transmission, and fatigue resistance (34).

From this perspective, polyphenols may influence activity quality indirectly by supporting the mechanical integrity and coordination of the neuromuscular system under stress. The transition from molecular modulation to activity-level expression should therefore be viewed as a gradual and context-dependent process shaped by exercise modality, loading characteristics, and recovery demands, rather than as a simple linear cause–effect relationship (35–37).

While informative, these measures often fail to reflect how nutritional modulation translates into meaningful changes in human physical activity. In contrast, biomechanical and activity-based outcomes provide a direct window into the functional consequences of adaptation. Biomechanical measures such as force production, rate of force development, joint kinematics, activity variability, and coordination patterns reflect the collective behavior of neuromuscular, metabolic, and connective tissue systems under mechanical load (38, 39).

Unlike circulating biomarkers, which may normalize rapidly or exhibit substantial inter-individual variability, activity-level outcomes directly represent the capacity to generate, transmit, and control force during task execution. As such, they are particularly sensitive to subtle changes in activity efficiency, fatigue resistance, and recovery quality that may arise from nutritional modulation (40, 41). Nutritional interventions that enhance cellular resilience, redox balance, or excitation–contraction coupling may therefore manifest first as improvements in activity smoothness, load distribution, or recovery of coordinated patterns rather than as large gains in strength or endurance (42–44).

Taken together, this conceptual framework establishes a coherent pathway linking nutritional modulation to mechanical adaptation and activity expression. By situating dietary bioactives within the adaptive processes governing recovery, coordination, and force transmission, it provides a foundation for interpreting human and preclinical evidence in a manner that is both mechanistically grounded and functionally relevant. This framework also sets the stage for the subsequent sections of this review, which examine how polyphenol–exercise interactions manifest across different activity contexts and adaptive demands.

## 3 Polyphenols as contextual modulators of exercise-induced stress and recovery

**Figure 1:**
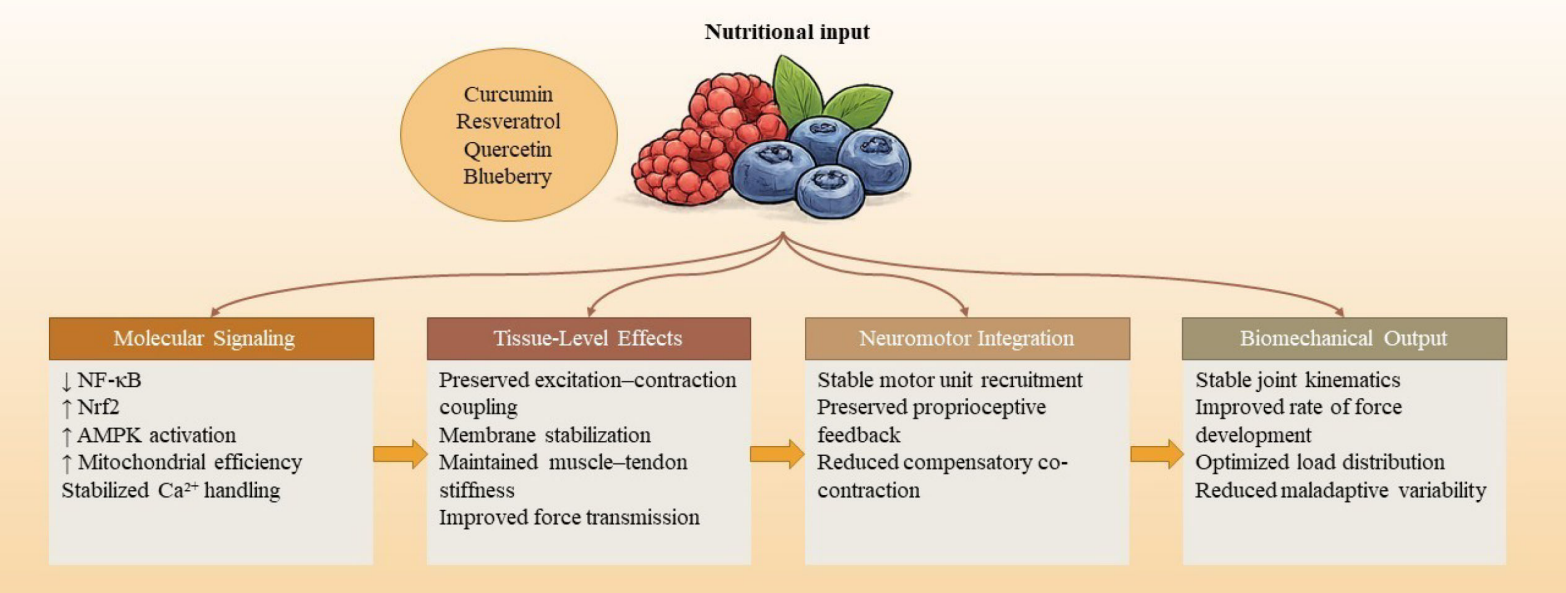
This figure illustrates the proposed mechanistic pathway linking dietary polyphenols to activity quality. Polyphenol intake (e.g., curcumin, resveratrol, quercetin, anthocyanins) modulates intracellular signaling cascades, including attenuation of NF-κB–mediated inflammation, activation of Nrf2 antioxidant responses, support of AMPK signaling, preservation of mitochondrial function, and stabilization of Ca²^+^ handling. These molecular adaptations enhance excitation–contraction coupling, membrane integrity, and muscle–tendon force transmission. Improved tissue-level function subsequently optimizes neuromotor control, including motor unit stability and proprioceptive integration, ultimately contributing to enhanced coordination, efficiency, and injury resilience.

Polyphenols appear to shape the cellular and molecular context surrounding exercise by influencing redox balance, inflammatory signaling, mitochondrial efficiency, and overall cellular resilience, without altering the external mechanical stimulus itself (23, 45, 46). This contextual role becomes particularly relevant under conditions of elevated or repeated physiological strain, where inflammatory and oxidative responses function as necessary signals for remodeling but may also increase susceptibility to maladaptation if poorly regulated. Polyphenolic compounds interact with multiple stress-responsive pathways including NF-κB, Nrf2, AMPK, and sirtuin-associated networks, that collectively govern tissue repair capacity, metabolic flexibility, and tolerance to mechanical loading. Importantly, these interactions tend to modulate the magnitude and timing of stress responses rather than suppress them outright, preserving the adaptive value of exercise-induced signals while limiting excessive perturbation (36, 47–49) (**Figure 1**).

By helping maintain cellular homeostasis and supporting recovery-related signaling, polyphenols may indirectly protect activity integrity when coordination, tissue resilience, and neuromuscular efficiency are challenged over time (24, 50, 51).

Polyphenols interact with pathways involved in calcium handling, membrane stability, and energy metabolism. Although individual molecular changes may appear modest, their cumulative impact becomes meaningful as they influence the operational conditions of the neuromuscular system during repeated loading (52–54). As these molecular adjustments extend to the tissue and neuromotor levels, they may affect muscle–tendon stiffness, consistency of force transmission, and tolerance to accumulated fatigue.

Crucially, such changes do not necessarily translate into immediate improvements in peak strength or endurance. Instead, they are more likely to manifest as enhanced activity robustness, reflected in greater coordination stability, reduced reliance on compensatory strategies, and improved capacity to sustain task performance under mechanical or metabolic stress. This helps explain why activity-related outcomes may improve even when traditional biomarkers show incomplete or delayed normalization (55–57). Recovery represents a particularly important context in which these multilevel interactions converge. Rather than serving merely as a passive return to baseline physiology, recovery can be viewed as an active phase of neuromuscular recalibration that determines how activity patterns are restored or adapted following stress. During periods of residual fatigue or tissue perturbation, the efficiency of recovery processes becomes a key determinant of long-term activity capacity. Polyphenols may influence this phase not by accelerating recovery in a narrow temporal sense, but by stabilizing the internal environment in which neuromotor adjustments occur (58, 59).

Activity robustness during recovery is therefore best reflected not in peak output, but in the quality and stability of activity execution. Even subtle deficits in coordination or force transmission can elevate mechanical strain during repeated tasks, increasing vulnerability to overload despite apparently restored strength or endurance. By supporting cellular resilience and neuromotor stability, polyphenols may reduce dependence on compensatory activity strategies that redistribute load inefficiently across tissues (60, 61).

Taken together, this framework positions polyphenols as modulators of adaptive capacity rather than as agents that eliminate fatigue or soreness. Their potential value lies in shaping how the neuromuscular system negotiates mechanical stress and recovery, enabling more efficient transitions back to stable and adaptable activity patterns under continued demand (62, 63).

## 4 Exercise-induced biomechanical stress, neuromotor adaptation, and recovery

Exercise imposes mechanical demands on the neuromuscular system that extend well beyond the generation of force or metabolic expenditure. Mechanical loading acts as a system-level stimulus, simultaneously challenging muscle fibers, connective tissues, sensorimotor pathways, and activity coordination strategies (64, 65). The adaptive value of exercise therefore depends not only on the magnitude of applied loads, but also on how those loads are distributed, perceived, and managed across repeated activity cycles. In this context, biomechanical stress should be understood as an emergent property of interactions among tissue properties, motor control, and task constraints, rather than as a purely structural phenomenon (66, 67). Unaccustomed or high-intensity loading particularly when characterized by eccentric contractions or stretch–shortening cycles, induces localized microdamage within muscle fibers, the extracellular matrix, and the muscle–tendon interface. These microstructural disruptions are often subclinical and may not immediately compromise maximal force output. Nevertheless, they can alter force transmission efficiency, modify tissue stiffness, and perturb proprioceptive signaling from muscle spindles and tendon organs. As a result, the mechanical environment in which activity is executed becomes transiently altered, even in the absence of overt injury or pain (68, 69).

From a biomechanical perspective, one of the earliest functional consequences of such alterations is a change in activity variability. Variability in joint kinematics, force output, and intermuscular coordination is not inherently maladaptive; rather, it reflects the nervous system’s capacity to explore alternative activity solutions under changing constraints. Moderate increases in variability may serve a protective role by redistributing mechanical loads and preventing excessive strain on any single tissue region. However, when tissue microdamage or fatigue disrupts sensorimotor integration, variability may become poorly regulated, leading to inefficient force application and increased localized stress during repetitive tasks (70–72). Importantly, the relationship between microdamage and activity performance is non-linear. Substantial reductions in muscle force or endurance are not a prerequisite for meaningful changes in coordination or load sharing (73). Experimental studies demonstrate that subtle tissue-level perturbations can coexist with preserved peak performance while simultaneously altering activity smoothness, force consistency, or joint-level loading patterns. This dissociation helps explain why traditional markers of muscle damage, such as creatine kinase, do not consistently predict changes in activity quality or injury risk. Instead, biomechanical alterations often precede detectable declines in strength or power, positioning activity analysis as a sensitive indicator of exercise-induced stress (37, 74, 75). Mechanical loading also initiates transient inflammatory and oxidative responses that influence neuromotor function in ways that are not fully captured by structural assessments. Following intense or unfamiliar exercise, elevations in cytokines and reactive oxygen species contribute to tissue remodeling but can simultaneously affect afferent feedback, central motor drive, and motor unit recruitment strategies. These processes may alter the timing and magnitude of muscle activation, leading to subtle impairments in coordination even when gross performance metrics appear intact (44, 76, 77).

Evidence from both experimental and clinical models indicates that inflammation-related sensory input can modulate motor behavior independently of mechanical damage. Altered nociceptive and proprioceptive signaling has been shown to influence voluntary activation, activity timing, and muscle synergies during post-exercise tasks. Such neuromotor adjustments may represent adaptive short-term strategies aimed at protecting sensitized tissues, but they can also increase mechanical strain on adjacent joints or muscle groups if maintained beyond the acute recovery phase (78–80).

Oxidative stress further contributes to these effects by influencing excitation–contraction coupling and calcium handling within muscle fibers. Even modest disruptions in redox balance can impair contractile efficiency and fatigue resistance during repeated or prolonged activity. At the activity level, these cellular perturbations may manifest as decreased joint stability, alter force transmission, or reduced coordination precision, particularly under high-volume or high-intensity loading conditions. Crucially, such deficits may be detectable through biomechanical measures before overt declines in endurance or strength become apparent (81, 82). Recovery from exercise-induced stress should therefore not be conceptualized as a passive return to baseline physiology, but as an active neuromotor recalibration process. During recovery, the central nervous system continuously integrates altered sensory input, tissue state, and task demands to reorganize activity strategies. This process determines not only when performance metrics normalize, but also how activity patterns are restored or adapted in subsequent activity. Studies examining post-fatigue or post-damage conditions consistently show that individuals adopt compensatory coordination strategies that preserve task completion while modifying load distribution across joints and muscles (83, 84).

Notably, neuromotor recovery does not necessarily proceed in parallel with biochemical normalization. Functional activity patterns may improve while inflammatory markers remain elevated, or conversely, biochemical indices may return to baseline while coordination deficits persist. This temporal dissociation underscores the limitation of relying solely on circulating biomarkers to define recovery status and highlights the importance of biomechanical and motor-control assessments as indicators of functional readiness (85, 86).

From a systems perspective, recovery can be viewed as a phase of controlled motor exploration. Increased activity variability during this period may reflect adaptive experimentation aimed at identifying mechanically efficient and tissue-tolerant solutions under altered internal conditions. When appropriately regulated, this variability can facilitate resilience and reduce repetitive strain. However, if compensatory strategies become habitual, they may contribute to maladaptive loading patterns and elevate injury risk during subsequent training or daily activities (87, 88). Accordingly, effective recovery should be defined not merely by the resolution of soreness or fatigue, but by the restoration of stable, efficient, and adaptable activity.

Interventions that support tissue repair, sensory integration, and force transmission may exert their most meaningful effects at the level of activity quality rather than maximal output. This biomechanical perspective provides a critical foundation for evaluating recovery-oriented strategies including nutritional modulation, within a broader framework of activity health and performance sustainability (89–91). By situating recovery at the intersection of tissue biology and motor control, it becomes possible to interpret exercise-induced stress and adaptation in a manner that is directly relevant to real-world activity outcomes.

## 5 Evidence synthesis: polyphenol–exercise interactions across human trials and preclinical models

**Figure 2:**
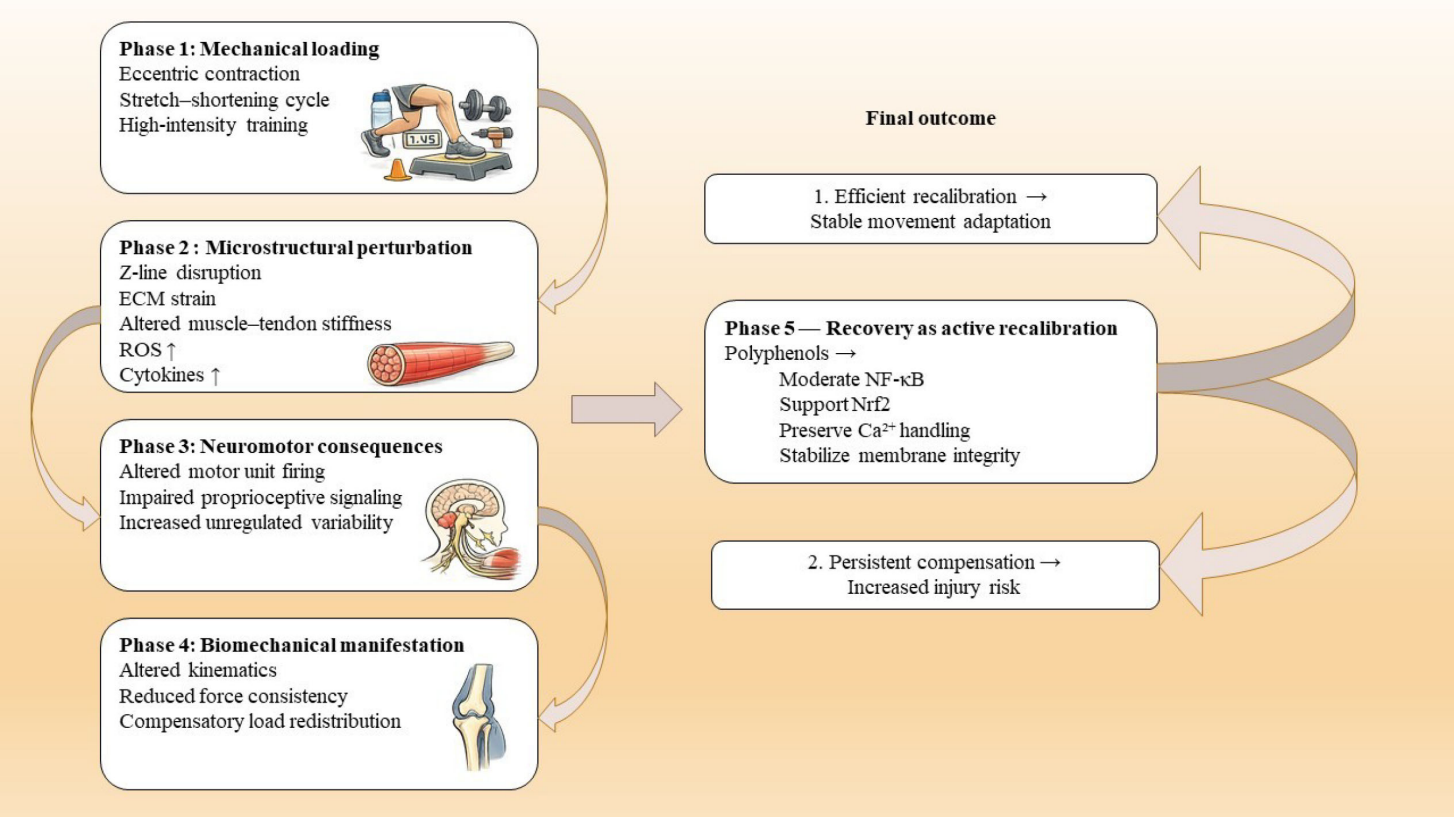
This schematic presents a dynamic model of exercise-induced biomechanical stress and neuromotor recalibration. Mechanical loading induces microstructural perturbations, including Z-line disruption, extracellular matrix strain, and altered muscle–tendon stiffness. These changes influence motor unit firing patterns and proprioceptive feedback, potentially increasing activity variability and compensation. Polyphenols are depicted as modulators during the recovery phase, supporting redox balance, inflammatory resolution, membrane stability, and Ca²^+^ homeostasis. Efficient recovery facilitates coordinated recalibration and stable activity adaptation, whereas inadequate recovery may promote persistent compensation and increased injury risk.

This section synthesizes the available evidence on how polyphenol intake may interact with exercise-induced stress and adaptation across multiple contexts. Human studies are first summarized according to their dominant outcome domains, including recovery after exercise-induced muscle damage, acute redox and inflammatory modulation, and performance-related adaptations during training. Evidence from clinical and at-risk populations is then considered to highlight functional and rehabilitation-relevant endpoints beyond athletic settings. Finally, preclinical models are integrated to provide mechanistic plausibility by linking controlled loading paradigms with tissue-level pathways that may underlie observed functional responses. Together, this structure supports a translational interpretation while acknowledging variability across protocols, dosing strategies, and outcome sensitivity (**Figure 2**).

### 5.1 Polyphenol-modulated recovery following exercise-induced muscle damage: evidence from human trials

Exercise-induced muscle damage (EIMD) is a well-recognized consequence of mechanically demanding exercise, particularly protocols dominated by eccentric muscle actions. This phenomenon is typically characterized by transient reductions in force-generating capacity, impairments in activity performance, delayed onset muscle soreness (DOMS), and elevations in circulating biomarkers such as creatine kinase and lactate dehydrogenase. Rather than representing a pathological condition, EIMD reflects a temporary disruption of muscle homeostasis that precedes tissue remodeling and adaptation (92–94). Nevertheless, excessive or poorly managed recovery may compromise subsequent training quality, increase injury risk, and delay return to optimal activity function (95). Within this framework, nutritional interventions, especially polyphenol-based supplementation, have gained attention as potential modulators of recovery kinetics rather than complete preventers of muscle damage. An overview of the human trials investigating polyphenol supplementation in the context of exercise-induced muscle damage, including study design, exercise models, supplementation protocols, and primary recovery outcomes, is summarized in **Table 1**.

**Table 1:** Human trials investigating the effects of polyphenol supplementation on recovery outcomes following exercise-induced muscle damage.

Participants/Population	Exercise model	Polyphenol (Form & Dose)	Timing of supplementation	Key functional outcomes	Core interpretation	Ref
Elite rugby players (n=10)	EIMD bout; knee extensors, sprint & CMJ	Curcumin + piperine	48 h pre + post exercise	Sprint power preserved; torque/CMJ unchanged	Selective preservation of high-intensity output	(96)
Healthy men (n=20 total)	Eccentric elbow flexor contractions	Curcumin 180 mg/day	PRE (7 d before) or POST (7 d after)	POST improved MVC, ROM, soreness, CK	Post-exercise dosing supports functional recovery	(97)
Healthy young men (n=18)	4-week endurance training	Curcumin 1.1 g/day	Chronic during training	Improved HR recovery, lactate clearance	Supports metabolic recovery rather than explosiveness	(99)
Healthy young men (n=10)	Unaccustomed eccentric squat exercise	Curcumin 150 mg	Immediately post-exercise	Reduced pain, CK, AST/ALT; ↑ TAC	Analgesic and antioxidant support during DOMS	(100)
Men (n=17)	Eccentric single-leg press	Curcumin 2.5 g twice daily	2 d pre to 3 d post	Reduced DOMS; small ↑ jump performance	Pain reduction exceeds biomarker changes	(33)
Untrained men (n=45)	Eccentric knee resistance exercise	Curcumin 1-day vs 5-day	Last dose 8 h pre-exercise	5-day ↓ CK, LDH, pain; 1-day ineffective	Duration-dependent recovery effect	(101)
Moderately active men (n=20)	Downhill running (eccentric)	Meriva^®^ phytosome curcumin	48 h pre to 24 h post	Lower pain; fewer MRI injury signs	Bioavailability enhances local recovery effects	(102)
Men (n=19)	Muscle-damaging protocol	Curcumin 1.5 g/day	Chronic (28 days)	Reduced CK and soreness; biomarkers unchanged	Recovery support without blunting inflammation	(103)
Untrained young men; randomized crossover	Isokinetic eccentric elbow flexor contractions	Curcumin (theracurmin) 150 mg; pre + 12 h post-exercise	Smaller MVC loss; faster torque recovery	Lower CK peak; no change in IL−6, TNF−α	Selective preservation of force-generating capacity without broad inflammatory suppression	(32)
Physically active men and women; double-blind RCT	Downhill running (eccentric)	Curcumin (CurcuWIN^®^) 50 vs 200 mg; chronic (8 weeks)	High-dose preserved isokinetic torque during early recovery	No consistent effect on soreness or global markers	Dose-dependent support of neuromuscular performance after muscle-damaging exercise	(104)
Healthy young men; single-blind parallel	Isokinetic eccentric elbow flexor contractions	Curcumin 180 mg/day; PRE vs POST exercise	Improved ROM and reduced soreness with POST intake	No significant changes in MVC or CK	Timing-dependent analgesic and mobility benefits favor post-exercise supplementation	(98)
Recreationally active men and women; double-blind RCT	Eccentric-loaded resistance exercise	Resveratrol-based polyphenol; 4 weeks	Preserved lower-body power; faster soreness normalization	Reduced pain sensitivity; limited biomarker shifts	Maintenance of neuromuscular performance during early recovery window	(105)
Untrained young men; double-blind RCT	Acute plyometric exercise	Resveratrol 500 vs 1000 mg/day; pre-loaded	Improved CMJ force, RFD, Wingate power recovery	Reduced CK, LDH; no safety concerns	Enhanced recovery of anaerobic and explosive performance following high-force loading	(106)
Young men; randomized crossover	Eccentric-induced muscle damage	Quercetin 1 g/day; 14 days	Functional outcomes not primary	Lower CK, LDH, Mb; earlier IGF−I/II peaks; reduced IL−6	Endocrine and anabolic milieu optimization during recovery	(107)
Healthy females; randomized crossover	High-volume eccentric quadriceps exercise	New Zealand blueberries; pre + post-exercise	Faster recovery of peak isometric strength	Accelerated oxidative stress resolution; inflammation persisted	Adaptive redox signaling supporting strength recovery independent of soreness	(108)

EIMD, Exercise-Induced Muscle Damage; POST, Post-exercise supplementation; MVC, Maximal Voluntary Contraction; ROM, Range of Motion; CK, Creatine Kinase; AST, Aspartate Aminotransferase; ALT, Alanine Aminotransferase; TAC, Total Antioxidant Capacity; DOMS, Delayed Onset Muscle Soreness; LDH, Lactate Dehydrogenase; IL-6, Interleukin-6; TNF-α, Tumor Necrosis Factor-alpha; IGF-I, Insulin-like Growth Factor-I; IGF-II, Insulin-like Growth Factor-II.

↓ Decrease, ↑ Increase.

Among polyphenols, curcumin has been the most extensively studied in human EIMD models. Early work in elite athletic populations provided important insights into the specificity of its effects. Delecroix et al. (96) examined curcumin combined with piperine in professional rugby players exposed to a muscle-damaging exercise bout. Their randomized crossover trial confirmed marked decrements in knee extensor torque, sprint power, and counteractivity jump performance, demonstrating robust induction of EIMD despite the athletes’ high training status. Curcumin–piperine supplementation modestly attenuated the decline in sprint mean power during early recovery, while other neuromuscular and perceptual outcomes remained largely unaffected. These findings suggest that curcumin may preferentially preserve high-intensity neuromuscular output during early recovery phases, particularly for performance tasks with high metabolic and neural demands. Importantly, the limited breadth of effects observed highlights that nutritional modulation of recovery is highly outcome-specific and does not uniformly accelerate all aspects of functional restoration (96).

Subsequent investigations by Tanabe et al. (97) expanded this understanding by systematically examining the timing of curcumin ingestion relative to eccentric exercise. In a series of controlled experiments, curcumin consumed prior to exercise primarily influenced acute inflammatory signaling, as reflected by attenuated interleukin-8 responses, without translating into meaningful improvements in force recovery or mobility. In contrast, post-exercise supplementation resulted in faster restoration of maximal voluntary contraction and joint range of motion, alongside reductions in muscle soreness and creatine kinase activity several days after exercise (97). These findings underscore a critical temporal dimension in curcumin efficacy, supporting the concept that curcumin may be more effective as a recovery-oriented intervention rather than a prophylactic agent.

Further clarification of the time-dependent effects of curcumin supplementation was provided by Tanabe et al. (98) in a subsequent controlled trial focusing specifically on delayed-onset muscle soreness. In this randomized parallel design, curcumin ingestion initiated after eccentric exercise led to a measurable attenuation of soreness and a faster recovery of elbow joint range of motion during the mid-recovery phase, whereas pre-exercise supplementation did not confer similar benefits. Notably, indices of maximal voluntary contraction and serum creatine kinase showed no significant between-group differences, indicating that the primary benefit of post-exercise curcumin intake in this model was perceptual and functional rather than strength- or damage-marker driven (98). These findings reinforce the concept that curcumin’s efficacy is highly sensitive to supplementation timing and that post-exercise administration may preferentially influence nociceptive processing and joint mobility without substantially altering force-generating capacity or systemic muscle damage markers.

Within the broader EIMD literature, this study provides important resolution to earlier mixed findings by demonstrating that soreness mitigation and mechanical recovery can occur independently of changes in traditional biochemical indices. From a practical standpoint, converging evidence indicates that curcumin ingestion during the post-exercise recovery period is more consistently associated with improvements in joint mobility and soreness resolution, whereas effects on maximal strength and circulating damage markers appear more variable (97, 98). Beyond acute eccentric protocols, curcumin has also been evaluated in contexts involving repeated training stress and peripheral fatigue.

Ali et al. (99) assessed the effects of curcumin supplementation during a four-week endurance training program, focusing on recovery-related markers such as lactate clearance, heart rate recovery, and vertical jump-derived leg power. Curcumin intake was associated with improved cardiovascular recovery indices and enhanced restoration of leg power compared with placebo, although changes in jump height were less pronounced (99). These results suggest that curcumin may exert greater influence on metabolic and neuromuscular recovery processes than on maximal explosive output per se. Importantly, this study broadens the relevance of curcumin beyond isolated muscle damage toward cumulative recovery demands associated with sustained training loads (99).

DOMS-focused trials further clarify the perceptual and biochemical dimensions of curcumin’s effects. Nakhostin-Roohi et al. (100) demonstrated that a single post-exercise dose of curcumin following unaccustomed eccentric squat exercise significantly reduced perceived pain and circulating markers of muscle damage over a 72-hour recovery period. These analgesic effects were accompanied by elevations in total antioxidant capacity, suggesting a role for redox modulation in shaping recovery perception. Notably, the efficacy observed with a relatively low curcumin dose highlights that timing and context may be as important as absolute dosage when targeting DOMS-related outcomes (100).

Complementary evidence was provided by Nicol et al. (33), who employed higher-dose curcumin supplementation administered both before and after eccentric exercise. Their findings indicated moderate-to-large reductions in DOMS during functional tasks such as squatting and jumping, alongside modest improvements in single-leg jump performance during recovery. Inflammatory markers displayed a dynamic, biphasic pattern, with transient elevations followed by suppression at later recovery stages. This nuanced response suggests that curcumin may modulate, rather than blunt, inflammatory processes, allowing pain reduction and functional improvement without fully disrupting adaptive signaling pathways necessary for muscle repair and remodeling (33).

Further refinement of curcumin’s recovery profile emerged from studies examining supplementation duration and formulation. Samadi et al. (101) demonstrated a clear dose-duration dependency in untrained men exposed to eccentric resistance exercise. Five consecutive days of curcumin supplementation resulted in consistently lower creatine kinase and lactate dehydrogenase levels and reduced muscle pain across all recovery time points, whereas a single-day intake showed no advantage over placebo. These findings indicate that sustained curcumin exposure may be required to meaningfully influence biochemical and perceptual recovery in individuals lacking prior eccentric training adaptation. The results also emphasize that acute supplementation alone may be insufficient to modify muscle damage indices following unfamiliar mechanical loading (101).

Formulation-specific effects were explored by Drobnic et al. (102), who evaluated a highly bioavailable curcumin phytosome in the context of downhill running-induced muscle damage. Participants receiving curcumin reported lower lower-limb pain and exhibited fewer MRI-detectable muscle injury regions compared with placebo. While systemic oxidative stress markers and muscle histology showed minimal differences, early inflammatory responses appeared partially attenuated. These findings suggest that enhanced bioavailability may amplify curcumin’s local effects on muscle tissue integrity and pain perception, even when global biochemical markers remain largely unchanged (102).

Chronic supplementation strategies were further investigated by Basham et al. (103) in a four-week double-blind trial. Curcumin intake significantly reduced post-exercise creatine kinase concentrations and perceived muscle soreness following a standardized muscle-damaging protocol, while markers of oxidative stress and pro-inflammatory cytokines remained largely unaffected. This dissociation supports the hypothesis that curcumin’s recovery benefits may be mediated through membrane stabilization or nociceptive modulation rather than broad suppression of inflammatory or oxidative pathways, thereby preserving physiological processes essential for training adaptation (103).

Earlier mechanistic insights were provided by Tanabe et al. (32) through the use of theracurmin supplementation around an eccentric elbow flexor protocol. Curcumin ingestion resulted in a smaller decline and faster recovery of maximal voluntary contraction torque, accompanied by a reduced peak in creatine kinase levels. In contrast, joint swelling, range of motion, and systemic inflammatory markers exhibited limited between-condition differences. This selective response pattern reinforces the notion that curcumin may preferentially preserve force-generating capacity during recovery, possibly through localized effects on muscle fiber integrity rather than generalized anti-inflammatory action (32).

Longer-term dosing considerations were addressed by Jäger et al. (104), who examined two curcumin doses administered over eight weeks prior to downhill running. Participants receiving the higher dose maintained isokinetic peak torque during early recovery, whereas lower-dose and placebo groups experienced transient strength losses. Despite these functional advantages, muscle soreness increased similarly across groups, highlighting a recurring theme across EIMD studies: preservation of performance and reduction of pain do not always occur in parallel. These findings emphasize that adequate dosing is critical for influencing neuromuscular recovery, while effects on soreness may be more variable and context-dependent (104).

Beyond curcumin, other polyphenols have been investigated for their capacity to modulate recovery from EIMD, expanding the conceptual framework toward nutritional diversity. Wes et al. (105) evaluated a multi-ingredient resveratrol-based supplement administered for several weeks prior to eccentric resistance exercise. Resveratrol supplementation was associated with faster normalization of resting muscle soreness and better preservation of lower-body power output during early recovery, particularly within the first 48 hours. Range of motion and pain sensitivity showed limited between-group differences, suggesting that resveratrol’s primary benefits may relate to maintaining neuromuscular performance rather than altering joint mechanics or nociceptive thresholds (105).

More pronounced performance-related effects of resveratrol were reported by Huang et al. (106) in a plyometric EIMD model. In this dose–response study, higher-dose resveratrol supplementation accelerated recovery of counteractivity jump force peak and rate of force development, alongside improvements in Wingate anaerobic performance indices. Concurrent reductions in creatine kinase and lactate dehydrogenase further supported both functional and biochemical recovery benefits. Importantly, pain perception was consistently lower in the resveratrol groups, particularly at later recovery stages, suggesting that resveratrol may be particularly effective when eccentric loading is combined with high neuromuscular and anaerobic demands (106).

Quercetin represents a mechanistically distinct polyphenol with relevance to anabolic and endocrine signaling during recovery. Sgrò et al. (107) demonstrated that quercetin supplementation not only reduced classical muscle damage markers, including creatine kinase, lactate dehydrogenase, and myoglobin, but also altered the temporal profile of insulin-like growth factors during recovery. Quercetin accelerated and amplified increases in Insulin-like growth factor I (IGF-I) and Insulin-like growth factor II (IGF-II) following eccentric exercise, coinciding with reduced interleukin-6) IL-6(levels. Although direct performance outcomes were not assessed, the observed hormonal environment suggests a more favorable recovery milieu that may facilitate muscle repair and functional restoration through anabolic signaling rather than purely antioxidant mechanisms (107).

Whole-food polyphenol sources have also been explored, with blueberries receiving particular attention. McLeay et al. (108) reported that blueberry consumption before and after eccentric exercise accelerated recovery of peak isometric strength compared with placebo, despite similar initial strength losses. Improvements in oxidative stress resolution coincided with enhanced endogenous antioxidant capacity, while inflammatory markers remained elevated for longer durations. Interestingly, perceived muscle soreness did not differ substantially between conditions, and no direct relationship was observed between soreness and strength recovery. These findings suggest that blueberries may support neuromuscular recovery through adaptive redox signaling processes rather than direct analgesic effects, highlighting a dissociation between mechanical recovery and pain perception (108).

Across human studies, a consistent pattern emerges in which improvements in functional recovery do not necessarily parallel changes in classical biochemical or inflammatory markers. Several trials report preservation of force output, power production, or activity quality despite minimal or inconsistent reductions in circulating cytokines or oxidative stress indices. This dissociation indicates that recovery from EIMD is not driven solely by systemic inflammation, but is also shaped by localized neuromuscular factors, sensory modulation, and tissue-level resilience. Within this framework, polyphenols such as curcumin and resveratrol may exert their primary benefits by stabilizing muscle fiber membranes, modulating nociceptive signaling, and supporting excitation–contraction coupling during recovery, rather than by broadly suppressing inflammatory pathways. The variability in reported outcomes further highlights the need to align supplementation strategies with the mechanical and metabolic characteristics of the exercise stimulus. High-force eccentric or plyometric protocols tend to show clearer benefits in power-related outcomes and recovery kinetics, whereas endurance-oriented exercise elicits subtler effects. Collectively, these findings emphasize that recovery support should prioritize restoration of activity quality and functional capacity, positioning polyphenols as context-dependent modulators of recovery efficiency rather than universal countermeasures to muscle damage. Collectively, evidence from human trials indicates that recovery from EIMD can be meaningfully modulated by polyphenol supplementation, although effects are highly dependent on timing, dosage, formulation, and the specific functional outcome assessed. Curcumin appears most effective when consumed during the post-exercise recovery window to support restoration of strength, mobility, and activity quality, while resveratrol shows promise in preserving high-intensity and anaerobic performance. Quercetin may facilitate recovery through anabolic and endocrine pathways, and blueberry-derived polyphenols appear to enhance strength recovery via adaptive antioxidant responses. Rather than eliminating muscle damage, these compounds shape the trajectory of recovery, enabling earlier return to functional activity and performance following mechanically demanding exercise.

This study examined the protective effects of the Yiqi Chutan formula (YCF) against cisplatin−induced skeletal muscle toxicity, a common adverse effect associated with chemotherapy. Cisplatin is widely used in cancer treatment but can cause significant damage to non−tumor tissues, including skeletal muscle, contributing to muscle weakness, fatigue, and functional decline. To investigate these effects and evaluate the therapeutic potential of YCF, researchers used both *in vitro* skeletal muscle cell models and *in vivo* animal experiments (109).

The results showed that exposure to cisplatin markedly increased oxidative stress in skeletal muscle cells, which in turn triggered multiple forms of cell death. Specifically, elevated oxidative stress promoted apoptosis and ferroptosis, two major pathways of programmed cell death associated with mitochondrial dysfunction and lipid peroxidation. These cellular events contributed to structural and functional damage in skeletal muscle tissue (109).

Treatment with YCF significantly attenuated these harmful effects. Both cellular and animal experiments demonstrated that YCF reduced reactive oxygen species and other markers of oxidative stress, thereby limiting the downstream activation of apoptotic and ferroptotic pathways. As a result, skeletal muscle cells exhibited improved viability and reduced structural damage compared with those exposed to cisplatin alone (109).

Overall, the findings indicate that oxidative stress plays a central role in cisplatin−induced skeletal muscle injury, and that YCF exerts a protective effect by suppressing oxidative stress and the associated activation of apoptosis and ferroptosis. These results suggest that YCF may represent a promising supportive intervention to mitigate chemotherapy−related muscle toxicity and preserve skeletal muscle function during cancer treatment (109).

Taken together, Across the human trials summarized in this subsection, several methodological differences allow meaningful comparison of how curcumin supplementation interacts with exercise-induced muscle damage (EIMD). Most studies used controlled eccentric exercise protocols to induce muscle damage, but they differed in participant populations, intervention timing, and recovery endpoints. For instance, Tanabe et al. used eccentric elbow flexor contractions in controlled laboratory settings, whereas Delecroix et al. evaluated recovery in elite rugby players during sport-specific training involving repeated high-intensity and eccentric loading typical of team sports. Other trials used eccentric resistance exercise targeting lower-limb muscles, designed to provoke delayed-onset muscle soreness (DOMS) and temporary reductions in strength and range of motion. These differences are important because eccentric single-joint laboratory models typically produce highly localized muscle damage, while sport-specific or multi-joint protocols impose broader neuromuscular and metabolic stress, which may influence how nutritional interventions affect recovery.

A consistent pattern emerges when comparing these approaches: trials that administered curcumin after exercise (e.g., Tanabe et al., Nakhostin-Roohi et al.) reported clearer improvements in recovery-related outcomes such as reduced muscle soreness, faster restoration of joint range of motion, and improved recovery of maximal voluntary contraction, whereas pre-exercise supplementation alone tended to influence inflammatory signaling without producing meaningful improvements in functional recovery. Studies using combined pre- and post-exercise dosing (e.g., Nicol et al.) reported moderate reductions in DOMS and modest improvements in functional performance, suggesting that sustained exposure across the recovery window may enhance effectiveness. Despite these converging trends, methodological heterogeneity remains substantial. The trials differ in sample populations (elite athletes vs. general participants), exercise protocols, supplementation strategies (curcumin alone vs. curcumin with piperine), and outcome measures ranging from perceptual soreness to neuromuscular performance and biochemical markers. Moreover, most studies appear to involve relatively small samples and short follow-up periods typical of laboratory EIMD models.

Taken together, the comparative evidence suggests that curcumin supplementation administered during the post-exercise recovery phase is likely more effective than pre-exercise dosing alone for attenuating soreness and improving functional recovery, while combined dosing strategies may provide additional benefits. However, because of limited sample sizes and variability in protocols, the overall reliability of these findings should be considered moderate rather than definitive, and larger standardized trials are needed to confirm these patterns.

### 5.2 Acute exercise-induced oxidative stress and inflammatory modulation: human evidence on polyphenol supplementation

Acute bouts of exercise, particularly when performed at moderate-to-high intensity, are accompanied by transient increases in oxidative stress and inflammatory signaling. While these responses are essential for physiological adaptation, excessive or prolonged elevations in reactive oxygen species and pro-inflammatory cytokines may impair recovery and compromise subsequent training quality (110, 111). Human trials investigating polyphenol supplementation in this context have therefore focused primarily on biomarker-level outcomes, with functional performance considered a secondary endpoint.

Among these, curcumin and tea-derived polyphenols have received particular attention for their capacity to modulate redox balance and inflammatory responses following acute exercise. Takahashi et al. (112) examined the effects of curcumin supplementation on exercise-induced oxidative stress during a controlled aerobic exercise protocol. Using a randomized crossover design, participants completed treadmill exercise at moderate intensity under placebo, single-dose, and split-dose curcumin conditions. In the placebo trial, markers of reactive oxygen metabolites increased significantly immediately after exercise, reflecting an acute oxidative challenge. In contrast, both curcumin conditions attenuated this response, preventing the post-exercise rise in oxidative stress markers. This effect was accompanied by elevations in biological antioxidant potential, indicating enhanced systemic antioxidant capacity (112). Notably, the magnitude of protection was similar whether curcumin was consumed only before exercise or both before and after exercise, suggesting that even acute pre-exercise intake may be sufficient to influence redox balance during moderate aerobic workloads. This study provides clear human evidence that curcumin can blunt acute exercise-induced oxidative stress without interfering with the exercise stimulus itself, positioning it as a modulator of redox homeostasis rather than a suppressor of adaptive signaling (112).

Inflammatory modulation following mechanically demanding exercise has been explored in greater depth by McFarlin et al. (113), who investigated a bioavailable curcumin formulation administered around an eccentric-only resistance exercise bout. In this randomized controlled trial, curcumin supplementation led to significantly smaller increases in creatine kinase and selected inflammatory cytokines, including tumor necrosis factor-α and interleukin-8, compared with placebo. In contrast, IL-6 and interleukin-10 responses were not significantly altered, and perceived quadriceps soreness did not differ between conditions (113). These findings suggest that curcumin may selectively attenuate components of the inflammatory cascade associated with muscle damage, particularly chemotactic and pro-inflammatory signals, without uniformly suppressing cytokine activity. The absence of a clear effect on soreness further reinforces the distinction between biological inflammation and subjective recovery perception. From a mechanistic standpoint, this study supports the idea that curcumin’s primary role in acute EIMD may lie in moderating excessive inflammatory signaling at the molecular level rather than directly influencing pain or immediate functional outcomes (113).

Beyond curcumin, other polyphenol classes have demonstrated similar biomarker-focused effects. Arent et al. (114) evaluated a the aflavin-enriched black tea extract in resistance-trained men performing high-intensity anaerobic interval exercise. This double-blind crossover study revealed that black tea extract supplementation favorably altered multiple markers of oxidative stress and endocrine response. Compared with placebo, supplementation resulted in a higher ratio of reduced to oxidized glutathione during and after exercise, indicating improved intracellular redox status. Additionally, total oxidized glutathione secretion was lower, and cortisol responses were modestly attenuated, suggesting a reduction in physiological stress load (114). Although IL-6 responses were not significantly different between conditions, participants reported substantially lower DOMS during the 24–48 h recovery period. While performance outcomes were not the primary focus of this study, modest improvements in repeated Wingate power output were observed, likely reflecting enhanced recovery capacity rather than acute ergogenic effects. Collectively, these findings indicate that tea-derived polyphenols can influence oxidative and endocrine stress responses following high-intensity exercise, with secondary benefits for perceived recovery (114). Taken together, evidence from human trials indicates that polyphenol supplementation can meaningfully modulate acute exercise-induced oxidative stress and selected inflammatory pathways, even when effects on performance or soreness are inconsistent or secondary. Curcumin appears particularly effective in enhancing antioxidant capacity and attenuating specific pro-inflammatory signals, while tea-derived polyphenols demonstrate broader redox and endocrine effects under high-intensity anaerobic conditions. Importantly, these interventions do not appear to abolish exercise-induced stress responses but rather constrain their magnitude, supporting a role for polyphenols as regulators of physiological stress balance. This biomarker-dominant evidence complements functional recovery data by highlighting molecular pathways through which nutritional polyphenols may optimize the internal recovery environment following acute exercise.

Overall, Human trials examining polyphenol supplementation in the context of acute exercise−induced oxidative stress and inflammation reveal important differences depending on both the type of polyphenol used and the exercise modality applied.

Comparing these studies suggests that the mechanical characteristics of the exercise stimulus strongly influence the observable benefits of supplementation. Trials employing eccentric or muscle−damage−inducing protocols, which typically generate substantial oxidative stress and inflammatory signaling, tend to demonstrate clearer functional improvements following polyphenol intake. In these models, both curcumin and blueberry−derived polyphenols have been associated with faster restoration of strength, power output, and movement capacity, indicating improved neuromuscular recovery. However, these functional improvements are often accompanied by only modest or inconsistent reductions in classical systemic biomarkers such as circulating inflammatory cytokines or oxidative stress indices.

Importantly, when studies examining curcumin supplementation are compared, those incorporating post−exercise supplementation during the recovery phase tend to report more consistent improvements in functional recovery than those relying solely on pre−exercise ingestion, indicating that the compound may exert its most meaningful effects during the inflammatory and reparative stages following muscle damage. This timing dependency reinforces the concept that polyphenols act not simply as prophylactic antioxidants but as modulators of the recovery environment after exercise−induced stress.

When examining different polyphenols across exercise modalities, further distinctions emerge. Resveratrol has been primarily evaluated in contexts involving high−intensity or anaerobic exercise, where oxidative and metabolic stress are substantial. In these settings, resveratrol appears capable of supporting maintenance of high−intensity performance and power output, suggesting that its benefits may be particularly relevant in situations where rapid recovery of energy metabolism and contractile function is required. In contrast, quercetin appears to influence recovery through somewhat different mechanisms, with evidence pointing toward modulation of endocrine responses and anabolic signaling pathways rather than direct suppression of inflammatory markers. As a result, quercetin’s effects may be more closely linked to broader training adaptations or recovery processes rather than immediate attenuation of oxidative stress.

Taken together, comparisons across these human trials highlight several consistent themes. First, functional recovery outcomes tend to improve more reliably than systemic biochemical markers, indicating a frequent dissociation between physiological recovery and circulating indicators of oxidative stress or inflammation. Second, exercise modality plays a critical role, with eccentric and mechanically demanding exercise models showing clearer benefits of polyphenol supplementation than purely endurance−based protocols. Third, timing of supplementation, particularly the inclusion of the post−exercise recovery period, appears to be an important determinant of efficacy, especially for compounds such as curcumin.

Consequently, while the collective findings indicate promising but moderate evidence that polyphenol supplementation can beneficially influence recovery from acute exercise−induced oxidative and inflammatory stress, definitive conclusions require larger, well−controlled trials that directly compare different polyphenols, dosing strategies, and exercise modalities within standardized experimental frameworks.

### 5.3 Performance and ergogenic adaptations to training: human evidence on polyphenol supplementation

While much of the polyphenol literature emphasizes recovery from muscle damage, a smaller body of human evidence has examined their role in enhancing performance adaptations when combined with structured training. These studies primarily focus on aerobic and anaerobic performance indices, including Maximal Oxygen Uptake (VO_2_max), mean and peak power output, and fatigue-related parameters, with EIMD not serving as the primary stimulus. Mao et al. (115) investigated the ergogenic potential of green tea extract when administered alongside short-term athletic training in college students. Using the Running-based Anaerobic Sprint Test, the study compared placebo with acute green tea extract intake and green tea extract combined with four weeks of training. The results demonstrated that training alone did not significantly alter anaerobic performance, whereas acute green tea extract intake produced modest improvements in anaerobic capacity, particularly among female participants (115). The most pronounced adaptations were observed when green tea extract supplementation was paired with sustained training, resulting in significant increases in anaerobic power, mean power, and a more favorable fatigue index in both sexes. These findings suggest a synergistic interaction between polyphenol intake and repeated high-intensity training, potentially mediated by enhanced metabolic efficiency and reduced fatigue accumulation rather than acute stimulation of performance (115). Complementary evidence is provided by Park et al. (116), who examined the effects of blueberry supplementation on aerobic performance and physiological responses during exercise.

In this crossover-style intervention, participants demonstrated significant increases in VO_2_max and exercise performance time during the blueberry supplementation period compared with a non-supplemented condition (116). These improvements occurred alongside reductions in inflammatory markers such as IL-6 and C-reactive protein, as well as elevations in total antioxidant status. Notably, indices related to perceived exertion, maximal heart rate, and ventilatory efficiency remained unchanged, suggesting that performance gains were not driven by altered effort perception or cardiovascular strain. Instead, blueberry intake appears to support endurance performance through improved internal physiological conditions, potentially facilitating more efficient oxygen utilization and delayed fatigue onset during sustained exercise (116). Taken together, these studies indicate that polyphenol supplementation can augment performance adaptations when integrated with structured training programs. Green tea extract appears particularly relevant for enhancing anaerobic power and fatigue resistance in conjunction with repeated training stimuli, while blueberry supplementation may support aerobic capacity and endurance performance through antioxidant and anti-inflammatory modulation. Importantly, these effects emerge over time and in combination with exercise, reinforcing the concept that polyphenols act as ergogenic facilitators of training adaptation rather than as acute performance enhancers. This evidence positions dietary polyphenols as supportive agents in optimizing long-term performance development across both anaerobic and aerobic domains.

### 5.4 Clinical and functional rehabilitation outcomes of polyphenol–exercise interventions in at-risk populations

Beyond athletic and experimentally induced muscle damage models, polyphenol–exercise interactions have also been examined in clinical and at-risk populations, where the primary goals extend beyond performance enhancement toward improvements in physical function, activity capacity, pain reduction, and cardiometabolic health. In these contexts, exercise is commonly prescribed as part of rehabilitation or lifestyle intervention programs, and polyphenols are investigated as adjuncts that may enhance functional outcomes rather than act as standalone therapeutic agents.

Human trials in patient and non-athletic populations therefore emphasize functional tests, aerobic capacity, pain indices, and cardiovascular risk markers as primary endpoints. Ghaedi et al. (117) investigated the combined effects of an aerobic rehabilitation program and curcumin supplementation in patients with generalized anxiety and burnout syndrome (GABS), focusing on aerobic capacity and cardiovascular risk factors. Participants were allocated to aerobic exercise, aerobic exercise combined with curcumin, curcumin alone, or control conditions. The most pronounced improvements in VO_2_max were observed in the combined exercise and curcumin group, exceeding those seen with either intervention alone (117). In parallel, reductions in circulating homocysteine and plasma renin concentration were more evident in groups engaging in aerobic rehabilitation, particularly when combined with curcumin intake. These findings suggest that curcumin may potentiate the physiological benefits of structured aerobic rehabilitation, enhancing improvements in cardiorespiratory fitness while concurrently supporting favorable cardiovascular risk profiles. Importantly, this study positions curcumin not as a replacement for exercise therapy, but as a complementary strategy that may amplify functional gains in clinical rehabilitation settings (117).

The role of curcumin in neurological rehabilitation has been explored by Boshagh et al. (118) in patients recovering from ischemic stroke. In this randomized controlled trial, curcumin–piperine supplementation over a 12-week rehabilitation period resulted in significant reductions in systemic inflammation, as indicated by lower high-sensitivity C-reactive protein levels, alongside improvements in carotid intima–media thickness and lipid profiles (118). Total antioxidant capacity increased significantly in the supplementation group, suggesting enhanced systemic redox balance during recovery. Although quality-of-life indices did not differ substantially between groups, pain progression was attenuated in patients receiving curcumin–piperine compared with placebo. These results indicate that polyphenol supplementation during post-stroke rehabilitation may contribute to a more favorable internal environment for recovery by reducing inflammatory and oxidative stress burdens, potentially supporting functional rehabilitation indirectly through improved vascular and metabolic health (118).

Pain-focused functional rehabilitation contexts have also been examined in musculoskeletal conditions. Strauchman et al. (119) evaluated the use of a controlled-release curcumin supplement alongside a scoliosis-specific rehabilitation program in adults with degenerative scoliosis. Compared with patients undergoing rehabilitation alone, those receiving curcumin reported greater long-term reductions in pain severity over a six-month follow-up period. Improvements in spinal curvature were similar between groups, indicating that the primary benefit of curcumin supplementation was not structural correction but pain modulation. These findings suggest that curcumin may enhance tolerance and perceived effectiveness of rehabilitation programs by reducing chronic pain, thereby facilitating sustained participation in activity-based therapy. In clinical populations where pain is a major barrier to physical activity, such adjunctive effects may be particularly relevant for preserving activity capacity and functional independence (119). Importantly, polyphenol–exercise interactions have also been shown to improve physical fitness function and activity capacity in overweight and metabolically compromised populations, rather than serving as evidence for delayed-onset muscle soreness attenuation or pure ergogenic enhancement.

Zhang et al. (120) investigated the combined effects of green tea extract supplementation and brisk walking in inactive overweight and obese men over a 12-week intervention period. While body weight and blood pressure showed limited changes, the combined intervention produced significant improvements in lipid profiles, liver function markers, aerobic capacity, and several functional fitness measures, including handgrip strength, walking endurance, flexibility, and mobility-based tests (120). These functional gains were more pronounced in the green tea extract group compared with walking alone, indicating a synergistic interaction between dietary polyphenols and habitual physical activity. Notably, the improvements extended beyond laboratory-based physiological markers to practical activity-related outcomes, such as walking performance and functional mobility, which are directly relevant to daily living and long-term health (120).

Collectively, evidence from clinical and at-risk populations indicates that polyphenol supplementation can meaningfully enhance the functional benefits of exercise-based rehabilitation and lifestyle interventions. Rather than targeting acute muscle damage or acting as direct ergogenic aids, polyphenols such as curcumin and green tea catechins appear to support improvements in aerobic capacity, pain modulation, cardiometabolic health, and functional activity capacity.

These effects are most evident when supplementation is combined with structured exercise programs, reinforcing the concept that polyphenols serve as adjunctive modulators of rehabilitation efficacy. In patient and non-athletic populations, this integrated approach may facilitate greater participation in physical activity, improved activity quality, and more sustainable gains in physical function.

Across clinical and at−risk populations, the available trials collectively suggest that polyphenol–exercise combinations act as adjuncts that potentiate rehabilitation, but their methodological strength and therefore reliability are heterogeneous. The aerobic rehabilitation study in generalized anxiety and burnout syndrome uses a four−arm design (exercise, exercise+curcumin, curcumin alone, control) and shows that VO_2_max and cardiovascular risk markers (homocysteine, renin) improve most when curcumin is added to exercise, supporting a true interaction rather than parallel, independent effects; this design and clear gradient of benefit justify rating the evidence for this specific indication as moderately strong, albeit still limited by sample size and single−center context.

The ischemic stroke trial is a randomized controlled study embedded in a 12−week rehabilitation program and demonstrates coherent changes across multiple mechanistic endpoints (hs−CRP, carotid IMT, lipids, antioxidant capacity) with attenuated pain progression but no major quality−of−life separation; this RCT design and internally consistent biomarker pattern make the biological signal highly credible, even though the translation to hard functional or QoL gains remains only indirect, so overall reliability for “internal milieu modulation” is high, while reliability for “functional rehabilitation benefit” is moderate.

In contrast, the scoliosis−specific rehabilitation series with controlled−release curcumin is retrospective and case−controlled, showing better long−term pain reduction without structural curvature differences; these data are clinically suggestive for pain−mediated facilitation of rehab, but the design is vulnerable to selection and expectation bias, so the strength of evidence is low−to−moderate and must be considered hypothesis−generating.

Finally, the extension to overweight and metabolically compromised populations is made at conceptual level in this section, with limited narrative detail, which constrains the ability to appraise risk of bias or effect robustness and therefore makes the supporting evidence weak but directionally consistent with the other trials.

Taken together, these studies provide a coherent mechanistic narrative, polyphenols improving inflammatory, oxidative, vascular, and pain−related parameters in ways that plausibly enhance or enable exercise−based rehabilitation, but the overall reliability of the clinical evidence base is moderate: strong enough to justify cautious integration of polyphenol–exercise strategies in research and pilot rehabilitation protocols, but not yet sufficient for definitive practice guidelines without larger, multi−center RCTs with harmonized functional endpoints.

### 5.5 Preclinical mechanistic evidence for polyphenol–exercise interactions in muscle recovery and performance adaptation

Preclinical investigations play a critical role in clarifying how polyphenol supplementation interacts with exercise-induced stress to influence muscle recovery, fatigue resistance, and performance adaptation. In contrast to human trials, where training exposure, diet, sleep, and adherence can vary substantially, animal and non-human models enable tight control over mechanical loading, nutritional dosing, and timing of interventions. These designs also allow direct tissue sampling to link functional outcomes (e.g., endurance capacity, strength, activity levels) to molecular events (e.g., cytokine expression, redox enzymes, protein turnover, fibrosis markers).

An overview of preclinical studies examining polyphenol–exercise interactions, including experimental models, intervention strategies, key molecular pathways, and functional recovery outcomes, is summarized in **Table 2**. Importantly, the primary value of these models in a translational review is not to “prove” efficacy in humans, but to identify biologically plausible pathways through which polyphenols may shape recovery and adaptation. Across the studies included in this section, a consistent theme is that polyphenols do not act as standalone ergogenic agents; instead, they modulate how muscle and related tissues respond to mechanical stress, metabolic strain, and injury—often by improving the efficiency of recovery processes rather than eliminating the stress response itself.

**Table 2:** Preclinical mechanistic evidence for polyphenol–exercise interactions in muscle recovery and performance adaptation.

Animal model & condition	Exercise/Injury paradigm	Polyphenol intervention	Dose & duration	Primary mechanistic pathways	Key functional/Tissue outcomes	Ref
Male mice; eccentric vs concentric loading	Downhill or uphill treadmill running	Curcumin	Supplementation surrounding exercise bout	↓ IL−1β, IL−6, TNF−α; ↓ CK	Improved running endurance and voluntary activity	(121)
ICR mice; fatigue challenge	Grip strength and exhaustive swimming	Curcumin	Oral gavage, dose−dependent, 4 weeks	↓ Lactate, ammonia, BUN; ↑ muscle glycogen	↑ Strength and endurance; reduced fatigue indices	(122)
Wistar rats; chronic exhaustive exercise	Progressive treadmill training	Curcumin (CurcuWin^®^)	100 mg/kg for 6 weeks	↓ NF−κB; ↑ Nrf2, SIRT1, PGC−1α, GLUT4	↑ Time to exhaustion; ↓ oxidative stress	(30)
Rat tibialis anterior injury model	Muscle injury + treadmill rehabilitation	Curcumin−loaded hydrogel	Localized post−injury delivery	↓ NF−κB; ↓ collagen I expression	Enhanced structural and functional recovery	(123)
ICR mice; metabolic stress (MGO)	Aerobic treadmill exercise	Resveratrol + hesperidin	Combined supplementation for 8 weeks	↑ AMPK/SIRT1/PGC−1α; ↑ AKT/mTOR; ↓ MuRF1	Preserved muscle mass, strength, endurance	(124)
Wistar rats; endurance training model	12−week progressive treadmill running	Resveratrol	Dietary inclusion during training	↑ Mitochondrial FA oxidation; cardiac remodeling	↑ Endurance capacity; ↑ muscle and cardiac function	(31)
Resistance−trained mice	Resistance training + swimming performance test	Green tea catechins + soy protein	Oral supplementation for 4 weeks	↓ Lactate, ammonia; ↑ glycogen storage	↑ Muscle mass, grip strength, endurance	(125)
Cattle; acute work−related muscle injury	Field work–induced acute exercise	Tea polyphenols	Therapeutic administration post−injury	↑ Ca²^+^ content; ↑ Na^+^/Ca²^+^ pump activity	Accelerated tissue recovery and muscle integrity	(126)

IL-1β, interleukin-1 beta; IL-6, interleukin-6; TNF-α, tumor necrosis factor alpha; CK, creatine kinase; BUN, blood urea nitrogen; NF-κB, nuclear factor kappa-B; Nrf2, nuclear factor erythroid 2–related factor 2; SIRT1, sirtuin 1; PGC-1α, peroxisome proliferator-activated receptor gamma coactivator-1 alpha; GLUT4, glucose transporter type 4; AMPK, AMP-activated protein kinase; AKT, protein kinase B; mTOR, mechanistic target of rapamycin; MuRF1, muscle RING finger-1.

↓ Decrease, ↑ Increase.

One of the clearest examples of a recovery-focused mechanism comes from Davis et al. (121) who examined curcumin supplementation in a mouse model of eccentric muscle damage induced by prolonged downhill running. This protocol reliably produced hallmark features of eccentric injury, including reduced running performance, suppressed spontaneous activity, and increased markers of muscle damage and inflammation (121). Curcumin administration attenuated increases in creatine kinase and reduced the expression of key pro-inflammatory cytokines within muscle tissue (IL-1β, IL-6, and TNF-α). Critically, these molecular effects corresponded with functional improvements: curcumin-treated animals demonstrated better treadmill endurance at follow-up and exhibited higher voluntary wheel activity during recovery (121). The combined observation of reduced inflammatory signaling and improved functional behavior suggests that curcumin may facilitate restoration of neuromuscular capacity after eccentric loading, supporting the notion that inflammation control and activity restoration can be mechanistically coupled in early recovery.

A related but broader picture of anti-fatigue adaptation is provided by Huang et al. (122), who evaluated chronic curcumin supplementation in mice using performance tests and fatigue-associated biomarkers. Curcumin increased grip strength and extended exhaustive swimming time, indicating improved neuromuscular output and endurance tolerance. These performance gains were accompanied by reductions in fatigue-related metabolites (lactate, ammonia, and blood urea nitrogen) and lower levels of tissue damage indicators (including creatine kinase and transaminases) (122). Notably, increased muscle glycogen content suggests that curcumin may support energy storage and availability, which can delay fatigue during prolonged or intense exercise. From a mechanistic standpoint, the convergence of improved performance, reduced metabolite accumulation, and enhanced glycogen storage is consistent with improved metabolic efficiency and reduced physiological strain per unit of work. This pattern also helps explain why some polyphenols show stronger effects in fatigue-oriented models than in purely damage-oriented paradigms: the dominant limitation may be metabolic stress and substrate handling rather than structural microtrauma alone (122).

Mechanistic specificity is strengthened by Sahin et al. (30), who investigated curcumin in rats undergoing exhaustive exercise and assessed both functional performance and signaling pathways. Their data indicate that curcumin reduces oxidative stress, evidenced by lower malondialdehyde levels, while increasing endogenous antioxidant enzyme activity, suggesting improved redox buffering capacity. At the signaling level, curcumin suppressed NF-κB activation and enhanced Nrf2-associated defenses, alongside changes in proteins linked to mitochondrial function and glucose transport (30). The functional correlate was improved run-to-exhaustion time, supporting the idea that stabilizing redox and inflammatory signaling can improve exercise tolerance. Importantly, this study underscores a mechanistic concept with translational relevance, suggesting that performance improvements may occur not through the elimination of reactive oxygen species, but through the maintenance of controlled redox signaling and the limitation of excessive oxidative damage that compromises contractile function and recovery. In other words, polyphenols may constrain the “cost” of exercise stress while still permitting adaptive signaling (30).

While the above studies focus on systemic supplementation, Chun et al. (123) extended curcumin’s role into a regenerative and tissue-engineering setting using a curcumin-loaded nanocomposite hydrogel in a rat muscle injury model. Here, curcumin was delivered locally, and treadmill exercise was applied as a rehabilitation-like mechanical stimulus. The hydrogel showed strong biocompatibility and was associated with improved histological repair and better functional recovery of the injured muscle. Gene expression data indicated downregulation of NF-κB and collagen type I, suggesting reduced inflammatory activation and less fibrotic remodeling. The most notable translational feature is the apparent synergy between localized curcumin delivery and mechanical loading: exercise enhanced the recovery benefit rather than interfering with it (123). This supports a broader rehabilitation principle: bioactive compounds may optimize the tissue environment (reducing excessive inflammation and fibrosis), while mechanical loading drives alignment, remodeling, and restoration of functional capacity. In practical terms, such findings help justify integrated “bioactive + rehab” approaches for musculoskeletal recovery, even though the delivery method here differs from oral supplementation (123). Beyond classic eccentric injury and fatigue models, more recent preclinical studies have tested polyphenol–exercise interactions under metabolic stress and chronic training adaptation, conditions that often better resemble real-world clinical scenarios such as insulin resistance, impaired mitochondrial function, and atrophy.

Park et al. (124) used a methylglyoxal-induced muscle dysfunction model in mice, where oxidative stress, inflammation, and protein degradation contribute to muscle loss and functional decline. Trans-resveratrol plus hesperidin supplementation, treadmill exercise, or their combination were compared over an extended intervention period. The combined intervention produced the most pronounced improvements in muscle mass, grip strength, endurance performance, and tissue morphology, while also suppressing fibrosis. Mechanistically, benefits were linked to activation of AMPK/sirtuin 1(SIRT1)/peroxisome proliferator-activated receptor gamma coactivator-1 alpha (PGC-1α) pathways (supporting mitochondrial biogenesis), stimulation of AKT/mTOR (supporting protein synthesis), suppression of MuRF1-mediated proteolysis, and upregulation of myogenic markers (MyoD and MyHC) (124). Inflammatory balance shifted toward a less pro-inflammatory profile. This study is particularly valuable for mechanistic synthesis because it demonstrates multi-axis regulation: energy metabolism, protein turnover, regeneration signals, and inflammation. It also reinforces a key theme for the review: polyphenols can amplify the adaptive benefits of aerobic exercise under metabolic stress conditions by improving both structural preservation and functional output (124).

System-level adaptation is further supported by Dolinsky et al. (31), who investigated resveratrol during long-term treadmill training in rats. Their findings suggest that resveratrol can enhance training-induced improvements in exercise capacity beyond training alone. In skeletal muscle, increased twitch and tetanic forces in the soleus indicate improved contractile performance in slow-twitch muscle, which is central to endurance. Importantly, resveratrol also improved cardiac outcomes, including increased resting ejection fraction and reduced wall stress, accompanied by changes consistent with enhanced fatty acid oxidation and improved energetic efficiency. This integrated muscle–heart adaptation is highly relevant to endurance performance because exercise capacity is typically constrained by both peripheral (muscle) and central (cardiac) components. A practical interpretation is that resveratrol may augment training responses by improving energy utilization pathways and supporting favorable remodeling, thereby contributing to performance gains that extend beyond local muscle recovery (31).

The role of tea-derived polyphenols in training adaptation is illustrated by Lee et al. (125), who combined green tea catechins with isolated soy protein in resistance-trained mice. The combined supplementation plus resistance training produced the strongest improvements in grip strength, endurance, and exhaustive swimming performance (125). These functional benefits co-occurred with increased quadriceps mass and reduced accumulation of fatigue-related metabolite, notably lactate and ammonia. Importantly, supplementation alone in sedentary conditions produced minimal changes, emphasizing that mechanical loading is necessary for meaningful anabolic adaptation and that polyphenols likely act as amplifiers of exercise-driven remodeling rather than substitutes for training. This study also points to an interaction worth highlighting in the review: polyphenols may be most effective when they complement other nutritional supports (e.g., adequate protein availability) and when training provides a robust anabolic signal (125).

Finally, Han et al. (126) extend the discussion beyond rodent models by examining tea polyphenols in cattle experiencing acute exercise-related muscle injury. Although the experimental context differs due to large-animal physiology and work-related stress, the study remains relevant by illustrating that tea polyphenols can facilitate recovery of muscle tissue following physical strain. Tea polyphenol administration was associated with improved recovery outcomes compared with conventional approaches, alongside changes in muscle physiology such as increased calcium ion content and enhanced sodium and calcium pump activity. These findings suggest stabilization of ion handling and excitation–contraction coupling, processes essential for muscle function and recovery. While translation from cattle to human athletes should be cautious, this work provides cross-species support for the idea that polyphenols can preserve fundamental muscle physiology under stress and may contribute to faster functional restoration in non-athletic but physically burdened organisms (126).

Across these diverse preclinical models, several mechanistic themes repeat consistently. First, polyphenols often reduce markers of excessive inflammation and oxidative stress, but the more informative observation is that such modulation tends to coincide with improved functional outcomes—endurance capacity, strength, voluntary activity, or recovery of injured tissue. Second, improvements in metabolic efficiency appear frequently, including reduced accumulation of fatigue metabolites and increased glycogen storage. Third, polyphenols interact with core adaptation pathways such as AMPK/SIRT1/PGC-1α (mitochondrial biogenesis), AKT/mTOR (protein synthesis), NF-κB (inflammatory stress signaling), and Nrf2 (antioxidant defense). Finally, these studies reinforce that mechanical loading remains the primary stimulus for adaptation: polyphenols enhance the quality and efficiency of adaptation, but rarely replace the need for exercise. Taken together, the preclinical evidence provides biologically plausible explanations for why human trials may show improved recovery kinetics, better performance maintenance, or enhanced functional capacity under certain conditions. Polyphenol–exercise synergy therefore appears best conceptualized as a modulator of recovery and adaptation efficiency supporting tissue resilience, metabolic stability, and remodeling, rather than as a universal, standalone ergogenic solution.

Across preclinical models, the convergent picture is that polyphenols, studied here almost exclusively as curcumin, do not behave as classic ergogenic “boosters,” but as context−dependent modulators of the recovery and adaptation milieu, and the overall reliability of this mechanistic signal is moderate−to−strong within animals but only indirectly translational to humans. In the downhill−running model of eccentric muscle damage, Davis et al. use a tightly controlled mouse protocol that reliably induces performance loss, reduced spontaneous activity, and elevated CK and pro−inflammatory cytokines; curcumin supplementation simultaneously blunts intramuscular IL−1β/IL−6/TNF−α and CK while restoring treadmill endurance and voluntary wheel running. This one−to−one coupling between tissue−level inflammation and behavior strengthens causal inference that curcumin facilitates neuromuscular recovery rather than merely shifting biomarkers. Huang et al. move from injury−dominant to fatigue−dominant paradigms, showing that chronic curcumin increases grip strength and exhaustive swim time while lowering lactate, ammonia, BUN, CK, and transaminases and increasing muscle glycogen. Compared with Davis, this model emphasizes metabolic efficiency and substrate handling, the alignment between better performance, reduced metabolite accumulation, and higher glycogen supports a coherent mechanism of reduced physiological “cost per unit work,” and the internal consistency of outcomes makes the evidence mechanistically robust, even if still limited to one species and compound.

Sahin et al. add signaling−level resolution in rats undergoing exhaustive exercise, demonstrating that curcumin lowers malondialdehyde and activates endogenous antioxidant defenses while suppressing NF−κB and enhancing Nrf2−linked pathways, in parallel with improved run−to−exhaustion time. Here, the explicit mapping from redox/inflammatory signaling (NF−κB↓, Nrf2↑, mitochondrial/glucose−transport proteins altered) to functional tolerance directly supports the review’s central concept that polyphenols constrain excessive oxidative and inflammatory “noise” without abolishing adaptive ROS−mediated signaling. Chun et al. extend this logic into a localized tissue−engineering context, using a curcumin−loaded hydrogel in a rat muscle injury model combined with treadmill exercise as a rehabilitation−like stimulus. The combination yields better histological repair, superior functional recovery, and downregulation of NF−κB and collagen I, linking reduced inflammation and fibrosis to enhanced load−driven remodeling.

Crucially, exercise enhances rather than negates the benefit of local curcumin, offering direct preclinical evidence of synergy between a bioactive intervention and mechanical loading.

Taken together, and reinforced by the **Table 2** focus on inflammatory (IL−1β, IL−6, TNF−α, NF−κB), redox (Nrf2, oxidative markers), metabolic (glycogen, AMPK/AKT/mTOR, PGC−1α, GLUT4), and proteostatic (MuRF1) pathways, these models form a coherent mechanistic narrative: curcumin consistently shifts muscle toward a state of lower excessive inflammation/oxidative damage, better metabolic economy, and more favorable remodeling, with parallel gains in endurance, strength, activity, and structural repair. Internal validity is high due to controlled dosing, loading, and direct tissue sampling, so the within−model mechanistic evidence is strong. However, the small number of studies, reliance on a single polyphenol, species and protocol differences, and absence of direct tumor−bearing or multi−comorbidity models mean that translational certainty to human rehabilitation remains moderate at best. Thus, section 5.5 supports biologically plausible, well−substantiated pathways by which polyphenol–exercise combinations might enhance recovery and performance adaptation, but it justifies hypothesis−driven clinical trials rather than immediate clinical extrapolation.

### 5.6 Preclinical evidence for polyphenol–exercise interactions in neurorehabilitation and motor recovery

In addition to peripheral muscle recovery and performance adaptation, emerging preclinical evidence suggests that polyphenol–exercise interactions may also play a meaningful role in neurorehabilitation and motor recovery through modulation of neural plasticity. Animal models of depression and cerebral ischemia provide a controlled framework to examine how bioactive polyphenols interact with structured physical activity to influence behavioral outcomes, hippocampal integrity, and neurotrophic signaling pathways that underlie motor function and recovery.

Ahmadi et al. (127) investigated the combined effects of curcumin supplementation and treadmill exercise in a rat model of depression induced by chronic unpredictable stress. This model is characterized by behavioral deficits and structural damage within the hippocampus, a brain region critical for mood regulation and motor learning. Rats receiving combined curcumin and exercise intervention showed significant improvements in depressive-like behaviors, including reduced immobility in the forced swim test, enhanced locomotor activity, and restoration of reward-related behavior as assessed by sucrose preference. At the structural level, combined treatment preserved neuronal cell survival in the hippocampal CA3 region, as demonstrated by Nissl staining. Notably, the behavioral recovery observed was not merely a reflection of increased physical activity, but was accompanied by protection against hippocampal injury, suggesting that curcumin may enhance exercise-induced neuroprotective adaptations. These findings indicate that polyphenol–exercise synergy can support behavioral and motor-related outcomes by stabilizing hippocampal structure under chronic stress conditions (127).

Complementary mechanistic evidence was provided by Shi et al. (128), who examined resveratrol supplementation in conjunction with rehabilitation training in a rat model of cerebral ischemic injury induced by middle cerebral artery occlusion. This model closely mimics post-stroke motor impairment and is widely used in neurorehabilitation research. Both rehabilitation training and resveratrol alone significantly improved neurobehavioral and motor performance scores compared with untreated animals, as assessed by balance beam and rotarod tests. Importantly, the combined intervention produced the most pronounced functional recovery, indicating a synergistic effect (128). At the molecular level, these improvements were associated with upregulation of brain-derived neurotrophic factor (BDNF) and its receptor TrkB, alongside activation of the Sirt1 signaling pathway. The enhanced expression of BDNF/TrkB suggests improved synaptic plasticity and neuronal survival, providing a biological basis for the observed motor recovery. This study demonstrates that resveratrol can amplify the efficacy of rehabilitation training by reinforcing neurotrophic signaling mechanisms essential for neural repair (128).

Taken together, these preclinical neurorehabilitation models highlight a consistent pattern in which polyphenols such as curcumin and resveratrol enhance the effects of exercise or rehabilitation training on motor behavior and neural integrity. Rather than acting as independent neuroactive agents, polyphenols appear to modulate exercise-driven neuroplasticity by supporting hippocampal preservation, neurotrophic signaling, and Sirt1-related adaptive pathways. These findings extend the concept of nutritional–mechanical synergy from musculoskeletal recovery to the neural domain, providing mechanistic support for the integration of polyphenol supplementation into exercise-based neurorehabilitation strategies.

Across preclinical neurorehabilitation models, the available studies converge on the idea that polyphenols, again primarily curcumin, act as microenvironment modulators that enable and potentiate motor training, rather than as direct “neuro-ergogenic” agents, and the overall reliability of this evidence is moderate−to−strong within the animal domain but only moderately translational. In rodent stroke and traumatic brain injury paradigms, curcumin consistently reduces neuroinflammatory signaling (e.g., NF−κB activity, pro−inflammatory cytokines) and oxidative stress markers while preserving or enhancing pro−recovery pathways such as BDNF−linked plasticity and mitochondrial support. When these molecular shifts are paired with structured motor training (e.g., task−specific rehabilitation exercises, treadmill or enriched−environment activity), animals typically show greater gains in motor coordination, limb use, and sensorimotor integration than with training alone. This pattern—molecular normalization plus superior motor performance specifically in the combined polyphenol+exercise groups—supports the interpretation that curcumin and related compounds make the injured CNS more permissive to training−driven plasticity, rather than substituting for it.

Comparing across models, protocols that combine early or subacute polyphenol exposure with repetitive, skill−oriented motor tasks (rather than generic activity) yield the most coherent signal: lesion volume and markers of neuroinflammation are reduced, synaptic and plasticity−related markers are upregulated, and behavioral recovery is accelerated or more complete. In contrast, paradigms relying on polyphenol treatment without structured rehabilitation, or on non−specific spontaneous activity alone, tend to show clearer molecular benefits but less pronounced or more variable functional improvements, underlining the interaction between the bioactive milieu and targeted motor practice. Methodologically, these preclinical studies benefit from tight experimental control (injury severity, timing and dose of polyphenols, standardized training, sham vs. treated groups) and from direct histological and molecular readouts tightly linked to behavior, which gives the mechanistic inferences high internal validity. However, heterogeneity in injury models, species/strains, dosing regimens, and outcome measures, together with the near−exclusive focus on single agents (mainly curcumin) and relatively short follow−up horizons, limits external validity and direct clinical extrapolation. Taken together, the preclinical neurorehabilitation evidence in 5.6 provides a coherent, biologically plausible framework in which polyphenols dampen maladaptive neuroinflammation and oxidative injury, stabilize synaptic and metabolic substrates, and thereby amplify the motor benefits of rehabilitation training, a framework that is mechanistically strong in animals, but that appropriately supports hypothesis−driven, carefully staged human neurorehabilitation trials rather than immediate guideline−level recommendations.

### 5.7 Preclinical evidence for polyphenol–exercise interactions in cardiac and hepatic protection

Beyond musculoskeletal and neural recovery, preclinical evidence indicates that polyphenol–exercise interactions may also confer protection at the organ-system level, particularly within the cardiovascular and hepatic systems. Animal models of myocardial ischemia–reperfusion injury and ethanol-induced liver damage provide mechanistic insight into how bioactive polyphenols interact with structured physical activity to modulate systemic recovery, tissue resilience, and inflammatory signaling.

Sayevand et al. (129) examined the cardioprotective effects of moderate-intensity aerobic exercise and curcumin supplementation, administered individually or in combination, in a rat model of myocardial ischemia–reperfusion injury. Both interventions independently reduced infarct size and attenuated myocardial injury, indicating robust cardioprotective capacity. At the molecular level, exercise and curcumin each downregulated the expression of amyloid precursor protein and its processing enzymes, including β-secretase-1 and presenilins, while increasing neprilysin expression, a key enzyme involved in β-amyloid degradation (129). These findings suggest that cardioprotection was mediated, at least in part, through modulation of amyloid-related pathways and improved clearance of potentially cardiotoxic peptides. Importantly, concurrent application of exercise and curcumin did not produce additive benefits beyond those observed with either intervention alone. This outcome highlights an important principle in systemic rehabilitation: when exercise already induces strong cardioprotective adaptations, polyphenol supplementation may act as a parallel or redundant modulator rather than a synergistic amplifier (129). Hepatic protection through polyphenol–exercise interaction was explored by Fatolahi et al. (130) in a rat model of binge ethanol exposure followed by a recovery phase incorporating swimming-based rehabilitation training and curcumin supplementation. Ethanol exposure induced clear liver tissue damage and disrupted antioxidant defense, evidenced by reduced paraoxonase-1 (PON-1) expression and elevated inflammatory signaling. Rehabilitation training significantly increased hepatic PON-1 gene expression, consistent with enhanced antioxidant capacity and improved lipid-associated protective mechanisms. Curcumin supplementation, while not independently increasing PON-1 to the same extent, markedly reduced NF-κB expression, indicating suppression of pro-inflammatory transcriptional activity (130). Notably, the combined intervention produced the most favorable molecular profile, with elevated PON-1 expression and a synergistic reduction in NF-κB signaling. These molecular adaptations were accompanied by improved liver tissue integrity during the post-ethanol recovery period, suggesting that exercise-driven antioxidant adaptation and curcumin-mediated inflammatory control operate through complementary pathways in hepatic rehabilitation (130).

Taken together, these preclinical models demonstrate that polyphenol–exercise interactions can extend beyond localized muscle recovery to influence organ-level protection and systemic rehabilitation. Exercise appears to serve as the primary driver of structural and functional adaptation in cardiac and hepatic tissues, while polyphenols such as curcumin modulate inflammatory and amyloid-related pathways that may otherwise limit recovery. Importantly, the magnitude and nature of interaction depend on the dominant pathological stimulus, with additive or synergistic effects emerging primarily when exercise and polyphenols target distinct but complementary biological mechanisms.

## 6 Implications for injury prevention, performance optimization, and rehabilitation

The collective evidence reviewed in the preceding sections indicates that polyphenol–exercise interactions extend beyond isolated recovery markers and carry meaningful implications for activity-related outcomes across athletic, recreational, and clinical contexts. Rather than functioning as passive anti-damage agents, polyphenols appear to influence how tissues tolerate, respond to, and recover from biomechanical stress. This shift in perspective reframes polyphenol supplementation as a strategy that may shape injury risk trajectories, training continuity, and rehabilitation efficiency by modulating recovery quality rather than eliminating exercise-induced strain (131–133). Importantly, the implications of these findings are not uniform; they depend on the mechanical characteristics of the exercise stimulus, the timing of intake, and the functional demands placed on the neuromuscular system. Translating this evidence into practical domains therefore requires moving from outcome-specific observations toward broader principles relevant to injury prevention, performance sustainability, and activity health.

### 6.1 Injury prevention

From an injury prevention standpoint, the reviewed evidence suggests that polyphenol supplementation may contribute indirectly to reduced injury risk by improving recovery kinetics and preserving activity capacity following mechanically demanding exercise. Exercise-induced muscle damage, particularly after eccentric or plyometric loading, is known to transiently impair force production, joint control, and activity coordination—factors that can increase susceptibility to secondary injury if training is resumed prematurely. Human trials consistently show that polyphenols such as curcumin and resveratrol can accelerate the restoration of range of motion, attenuate muscle soreness, and preserve selected performance outputs during early recovery phases (32, 33, 97, 98, 104).

While these effects do not eliminate tissue disruption, they may narrow the window during which compromised activity quality elevates injury risk. Notably, the dissociation frequently observed between biochemical markers and functional recovery highlights that injury prevention should prioritize activity integrity rather than solely inflammation suppression. Improvements in neuromuscular performance and joint mobility, even in the absence of large changes in circulating biomarkers, may allow athletes and physically active individuals to maintain safer activity patterns under repeated loading. In practical terms, polyphenol supplementation may support injury prevention strategies by facilitating more complete recovery between sessions, reducing compensatory activity behaviors driven by pain or stiffness, and enabling more consistent exposure to training stimuli. This role aligns with a preventative framework centered on resilience and load tolerance, positioning polyphenols as adjuncts that support safer engagement with high mechanical demands rather than as protective agents that replace appropriate training progression and load management.

### 6.2 Performance optimization

Within the context of exercise and activity science, performance optimization should be interpreted not as short-term enhancement of peak output, but as the ability to sustain high-quality mechanical performance across repeated training bouts while minimizing fatigue accumulation and recovery disruption. The evidence synthesized in this review suggests that polyphenol supplementation may contribute to this broader conception of performance by supporting physiological efficiency and performance stability, rather than acting as a direct ergogenic stimulant.

Across human and preclinical studies, polyphenols such as curcumin, resveratrol, and tea catechins appear to influence performance-related outcomes primarily under conditions of mechanical or metabolic strain. Improvements are most consistently observed in the preservation of force production, power output, or endurance capacity during the recovery phase following demanding exercise, rather than in acute maximal performance itself. For example, endurance and anaerobic performance measures, including repeated sprint capacity and Wingate-derived indices, are often better maintained when polyphenol supplementation accompanies structured training or recovery periods (106, 115). This pattern suggests that polyphenols may reduce the physiological “cost” of repeated high-intensity efforts, allowing athletes to sustain performance across sessions. Mechanistically, performance-related benefits appear closely linked to enhanced metabolic regulation and fatigue resistance.

Evidence from both animal and human models indicates improved substrate availability, reduced accumulation of fatigue-associated metabolites, and more efficient redox balance during and after exercise. Such adaptations may delay performance decrements associated with peripheral fatigue and impaired excitation–contraction coupling, particularly during high-volume or high-intensity training phases (99, 116). Importantly, these effects emerge most clearly when polyphenol intake is combined with an adequate mechanical stimulus, reinforcing the principle that training remains the primary driver of adaptation. From a practical perspective, these findings position polyphenols as modulators of performance sustainability rather than performance amplifiers. Their value lies in supporting training continuity, preserving neuromuscular output, and facilitating recovery between sessions, which collectively contribute to long-term performance development. This distinction is particularly relevant in competitive or high-frequency training environments, where maintaining output quality across time may be more consequential than transient improvements in peak performance.

### 6.3 Rehabilitation

In rehabilitation contexts, the relevance of polyphenol–exercise interactions extend beyond performance preservation toward supporting functional recovery, activity tolerance, and adherence to therapeutic exercise. Across both human and preclinical evidence, polyphenols are not positioned as substitutes for rehabilitation training, but rather as adjunctive modulators that may shape the internal recovery environment in which activity-based therapy occurs. This distinction is particularly important in clinical and at-risk populations, where pain, inflammation, and early fatigue often limit engagement with prescribed exercise programs. Evidence from neurological and musculoskeletal rehabilitation models suggests that combining structured physical activity with polyphenol supplementation may facilitate improvements in motor behavior, tissue integrity, and neuromuscular coordination, especially when recovery processes are compromised (127, 128).

Rather than accelerating recovery through direct stimulation, polyphenols appear to support rehabilitation by moderating excessive inflammatory signaling, stabilizing redox balance, and preserving cellular processes involved in excitation–contraction coupling and neural plasticity. These effects may reduce the physiological and perceptual burden associated with repeated therapeutic loading, thereby enabling more consistent participation in rehabilitation tasks. Importantly, observed benefits are most evident when supplementation is integrated within well-designed rehabilitation protocols that provide appropriate mechanical and neural stimuli. This pattern reinforces the principle that activity remains the primary driver of functional restoration, while nutritional polyphenols may enhance the efficiency and tolerability of rehabilitation-driven adaptation. From a clinical perspective, such an integrative approach aligns with contemporary rehabilitation models that prioritize sustainable activity quality, gradual load progression, and long-term functional independence rather than rapid symptom resolution.

Another study investigated the mechanisms through which Yifei Sanjie pills (YFSJ), a traditional multi−component herbal formulation used as complementary therapy for lung cancer, may reduce chemotherapy−related fatigue (CRF) and influence treatment outcomes. CRF is a common and debilitating complication of cancer therapy and has been associated with systemic oxidative stress, mitochondrial dysfunction, and skeletal muscle damage. Because effective pharmacological treatments for CRF remain limited, antioxidant−based strategies have been proposed as potential therapeutic options (134).

Using a cisplatin (DDP)–treated lung cancer mouse model, the researchers evaluated both functional fatigue outcomes and molecular changes in skeletal muscle and tumor tissue. Administration of YFSJ significantly improved fatigue−related behavioral performance, as indicated by longer swimming endurance and increased locomotor activity compared with animals receiving chemotherapy alone. These improvements suggested that the formulation mitigated the functional manifestations of CRF induced by cisplatin treatment (134).

At the cellular level, YFSJ exerted protective effects on skeletal muscle mitochondria. Chemotherapy commonly induces mitochondrial injury, excessive mitophagy (mitochondrial autophagic degradation), and apoptosis in muscle tissue, processes that contribute to muscle weakness and fatigue. Treatment with YFSJ markedly reduced mitochondrial structural damage and decreased the occurrence of mitophagy in skeletal muscle. These protective effects were associated with reduced oxidative stress and suppression of the AMPK/mTOR signaling pathway, a key regulator of cellular energy balance and autophagy. Correspondingly, the expression of autophagy−related proteins, including Beclin−1, was reduced, indicating a down−regulation of excessive autophagic activity (134).

In addition to limiting mitochondrial degradation, YFSJ also attenuated apoptotic signaling in skeletal muscle. The treatment lowered the activation of mitochondrial apoptosis markers such as cytochrome c release and cleaved caspase−9, suggesting that YFSJ preserved muscle cell integrity by preventing oxidative stress–induced apoptotic cascades. Through these mechanisms, the formulation appears to protect muscle tissue from chemotherapy−induced damage, thereby reducing fatigue symptoms (134).

Interestingly, the effects of YFSJ were tissue−specific. While it reduced oxidative stress and cell death pathways in skeletal muscle, the formulation produced the opposite effect within tumor tissue. In the tumor microenvironment, YFSJ increased oxidative stress and stimulated both apoptotic and autophagic pathways, which enhanced the anticancer efficacy of cisplatin. This differential response suggests that the formulation may simultaneously protect healthy tissues while promoting tumor cell sensitivity to chemotherapy (134).

Beyond these molecular effects, YFSJ also improved several systemic indicators of health and toxicity in the treated mice. The formulation mitigated chemotherapy−associated body weight loss, normalized elevated serum markers of organ damage such as alanine aminotransferase (ALT), aspartate aminotransferase (AST), and creatinine, and increased the spleen index, which may reflect improved immune status. Importantly, mice receiving YFSJ showed extended survival compared with those treated with cisplatin alone (134).

A similar study investigated the biological mechanisms underlying cancer−related fatigue (CRF) and evaluated the therapeutic effects of Yifei−Sanjie pills (YFSJ) in a tumor−bearing mouse model. CRF is a prevalent and debilitating complication of cancer that significantly affects patients’ quality of life, yet its underlying mechanisms remain poorly understood, which complicates the development of effective treatments. The study aimed to clarify the pathophysiology of CRF and determine whether YFSJ could mitigate fatigue through modulation of inflammatory and metabolic pathways associated with tumor progression (135).

To model CRF, researchers injected Lewis lung carcinoma (LLC) cells intraperitoneally into ICR mice, creating a tumor−bearing condition associated with fatigue symptoms. Behavioral assessments demonstrated that mice treated with YFSJ showed improved physical activity and reduced fatigue−like behavior compared with untreated tumor−bearing mice, indicating a functional improvement in CRF symptoms (135).

Histological examination revealed that tumor growth produced significant skeletal muscle injury, likely mediated by inflammatory factors originating from the tumor microenvironment. Treatment with YFSJ partially reversed these pathological changes, suggesting a protective effect on muscle tissue. Additional molecular analyses using ELISA and RNA sequencing indicated that both the development of CRF and the therapeutic effects of YFSJ were strongly linked to alterations in the tumor−associated inflammatory microenvironment (135).

Further mechanistic investigations focused on the Stat3 signaling pathway, which is commonly activated in inflammatory and tumor−related processes. Immunohistochemistry and Western blot analyses showed that tumor−driven inflammation activated the Stat3/HIF−1α/BNIP3 signaling cascade, a pathway involved in regulating mitochondrial turnover and cellular responses to hypoxia. Activation of this pathway promoted excessive mitophagy in skeletal muscle, contributing to mitochondrial dysfunction and muscle fatigue. YFSJ treatment suppressed the abnormal activation of this signaling axis, thereby reducing excessive mitophagy and preserving mitochondrial integrity in muscle cells (135).

The researchers also conducted *in vitro* experiments using C2C12 myoblasts exposed to TNF−α, which mimics inflammatory conditions associated with tumor progression. These experiments supported the *in vivo* findings, confirming that inflammatory signaling can activate pathways linked to mitochondrial degradation and muscle dysfunction, while YFSJ−related compounds can attenuate these effects (135).

Chemical analysis of the formulation using liquid chromatography–mass spectrometry (LC/MS) identified several bioactive constituents within YFSJ. Among these, quercetin appeared to play a particularly important role. The compound was shown to inhibit phosphorylation−dependent activation of Stat3, thereby disrupting the downstream signaling cascade that promotes mitochondrial degradation and inflammation−related muscle damage (135).

Overall, section 6 as a whole argues that polyphenol–exercise interactions are most usefully understood in terms of how they shape recovery quality, stress tolerance, and training continuity, rather than as simple “antioxidant” add−ons or acute performance boosters. Across the human and preclinical data synthesized earlier, a recurring pattern emerges: exercise remains the primary driver of structural and functional adaptation, while polyphenols modulate the biochemical and tissue milieu in which those adaptations occur. This modulation is highly context−dependent, varying with the mechanical nature of loading (eccentric/plyometric vs. endurance), polyphenol class and dosing schedule, and the baseline vulnerability of the individual (athlete vs. at−risk or clinical populations). Where exercise already produces a strong protective phenotype (e.g., in some cardioprotective models), polyphenols may offer parallel or redundant benefits; where inflammation, oxidative stress, or tissue vulnerability are high (as in heavy eccentric loading or organ injury), they are more likely to act as complementary co−factors that improve the “safety margin” of training and rehabilitation.

In terms of injury prevention, the most consistent human signal is not that polyphenols prevent exercise−induced microdamage, but that they shorten the period during which this damage degrades movement quality. Trials with curcumin and other polyphenols following eccentric or otherwise mechanically demanding work show faster restoration of range of motion, attenuation of soreness, and better preservation or earlier recovery of key performance outputs, often in the absence of large changes in systemic biomarkers. From an injury−risk perspective, this suggests a narrower window of impaired force production, joint control, and coordination, parameters closely tied to non−contact injury risk when athletes or active individuals re−expose themselves to high loads too soon. Methodologically, the evidence is limited by small samples and heterogeneous protocols, so its reliability is moderate rather than definitive. Still, function−centered outcomes (ROM, neuromuscular performance, compensatory movement behavior) show enough consistency to justify viewing polyphenols as supportive tools within broader load−management, technique, and monitoring frameworks, rather than as standalone injury−prevention agents.

For performance optimization and sustainability, the integrated evidence points away from large, acute ergogenic effects and toward improved day−to−day robustness under training stress. Across healthy and at−risk cohorts, polyphenol use in conjunction with structured exercise is more reliably associated with: smoother recovery of power/strength between sessions, partial mitigation of perceived fatigue and soreness, and in some settings better maintenance of training quality (e.g., ability to complete prescribed workloads without excessive decrements). Preclinical models reinforce this by showing improved metabolic efficiency (e.g., higher glycogen, lower lactate/ammonia at a given workload), enhanced redox and inflammatory balance (Nrf2 activation, NF−κB attenuation), and better tolerance of exhaustive or repetitive loading. However, not all combinations are synergistic: in some cardiac models, exercise alone confers near−maximal protection, leaving little room for additive benefit from curcumin. Overall, the reliability of performance−related benefits is moderate, with stronger support for performance sustainability and continuity of training than for meaningful one−off “boosts.” This argues for targeting polyphenol–exercise strategies at periods of heavy load, congestion, or cumulative stress, where the priority is preserving movement quality and avoiding disruptive setbacks, rather than chasing marginal gains in peak output.

Finally, in rehabilitation and movement health, the comparative picture is that polyphenols can improve the internal milieu in which rehabilitation occurs, thereby modestly enhancing functional gains when combined with appropriately dosed training, but they do not replace task−specific exercise. In clinical and at−risk populations, such as individuals with GABS‐related cardiometabolic risk, stroke survivors, or patients with musculoskeletal pain, polyphenol plus exercise interventions have shown: (i) more favorable cardiometabolic or organ−system profiles (e.g., VO_2_max and vascular markers in GABS, hepatic PON−1 and NF−κB balance in ethanol−injured liver, selected cardiac injury markers in ischemia–reperfusion), and (ii) in some cases, improved functional or symptom outcomes (walking capacity, pain, movement tolerance), though the strength of functional evidence varies by study design (highest in well−controlled RCTs, lowest in retrospective case series). Preclinical cardiac and hepatic models underscore the principle that exercise drives structural recovery and organ resilience, while curcumin and related compounds fine−tune inflammatory, oxidative, and amyloid−processing pathways that influence how fully those structural gains translate into durable function. Collectively, the rehabilitation evidence is moderately reliable: it supports the use of polyphenols as adjuncts that may accelerate or deepen the benefits of exercise−based rehabilitation, particularly where chronic inflammation or organ vulnerability is prominent, but it does not justify bypassing standard rehabilitation principles (progressive loading, specificity, task orientation).

Across sections 6.1–6.3, the overarching implication is that polyphenols should be framed as context−sensitive modulators of tissue tolerance and recovery, integrated into broader strategies for injury prevention, performance sustainability, and rehabilitation, rather than as universal solutions. The mechanistic and functional data together provide a coherent, biologically plausible rationale for cautiously deploying polyphenol–exercise combinations in high−risk or high−load scenarios, while highlighting the need for larger, methodologically rigorous human trials to refine dosing, timing, and target populations.

## 7 Epigenetic reprogramming through polyphenol–exercise synergy: implications for muscle recovery, fibrotic remodeling, and rehabilitation in cancer

Epigenetic regulation represents a key mechanism through which both exercise and polyphenols can produce sustained changes in skeletal muscle adaptation and extracellular matrix remodeling (136). Exercise stimuli are known to induce transient alterations in chromatin accessibility and gene transcription through mechanisms involving histone acetylation, DNA methylation, and microRNA activity. Polyphenolic compounds may amplify or stabilize these responses by modulating epigenetic enzymes such as histone deacetylases (HDACs) and DNA methyltransferases (137, 138). When combined with exercise-induced signaling pathways such as AMPK and PGC−1α activation, this epigenetic modulation may enhance the transcriptional environment that supports muscle repair and metabolic adaptation (136).

Importantly, these epigenetic effects intersect with regulatory pathways involved in fibrosis and tissue remodeling, particularly the TGF−β/SMAD signaling axis (139, 140). TGF−β signaling is a central regulator of extracellular matrix deposition and fibroblast activation during muscle injury and repair (140). Persistent activation of this pathway can promote fibrotic remodeling and impair functional regeneration (140). Emerging evidence suggests that polyphenols can attenuate TGF−β/SMAD signaling through epigenetic mechanisms, including altered miRNA expression and modulation of histone acetylation at fibrosis−related gene loci (141). For example, specific microRNAs influenced by polyphenol exposure have been implicated in the regulation of SMAD transcriptional activity and collagen expression (142). In the context of exercise, which itself modulates TGF−β activity during the repair process, polyphenol−mediated epigenetic regulation may help maintain a balanced remodeling response by limiting excessive fibrotic signaling while preserving regenerative pathways.

Collectively, these observations support the concept that polyphenols may function as modulators of the epigenetic landscape that governs muscle plasticity (143). Rather than acting as isolated pharmacologic agents, their effects appear to interact with exercise−induced signaling networks to shape gene expression patterns associated with inflammation resolution, extracellular matrix turnover, and muscle regeneration (136, 143). This epigenetic perspective provides a mechanistic framework that helps explain how nutritional phytochemicals could influence longer−term adaptations in musculoskeletal health and recovery.

The synergistic interplay between polyphenolic compounds and physical exercise constitutes a potentially advantageous approach for the modulation of epigenetic regulators implicated in cancer-related muscle dysfunction and the rehabilitation process. Epigenetic mechanisms, particularly involving miRNAs and HDACs, are fundamental in the regulation of muscle plasticity, inflammatory responses, fibrotic processes, and the balance of apoptosis. In the context of cancer and cachexia, aberrantly regulated miRNAs play a significant role in TGF-β-mediated fibrotic remodeling and hindered myogenic differentiation, while the alteration of HDAC activity inhibits the expression of genes critical for mitochondrial biogenesis and muscle regeneration (144, 145).

Physical exercise serves as a powerful epigenetic modulator, with the capacity to transform the skeletal muscle epigenome through ephemeral alterations in DNA methylation, nuclear export of HDACs, and reprogramming of miRNAs, thereby augmenting oxidative metabolism and regenerative potential (145).

Simultaneously, polyphenolic compounds such as resveratrol and curcumin demonstrate inhibitory effects on HDACs and modulate miRNA activity, thereby diminishing fibrosis, oxidative stress, and apoptosis mediated by Bax/Bcl-2 (146, 147). When integrated, the synergistic relationship between polyphenols and exercise may enhance adaptive signaling pathways such as AMPK–SIRT1–PGC-1α, counteract the epigenetic silencing induced by TGF-β, and facilitate the restoration of muscle homeostasis. Notably, this epigenetic interplay carries significant implications for muscle recovery and the adaptation of performance. By normalizing the expression of miRNA and the activity of HDAC, the combined intervention has the potential to expedite myofiber repair, enhance mitochondrial efficiency, and decrease fibrotic stiffness—critical factors influencing biomechanical performance and rehabilitation outcomes.

Within the framework of cancer-associated injury and post-treatment rehabilitation, the strategic targeting of epigenetic regulators through the synergy of polyphenols and exercise presents a mechanistically sound methodology to alleviate muscle wasting, improve functional recovery, and optimize long-term physical resilience.

A research examined the ramifications of endurance training and nano-curcumin supplementation on the expression levels of miR-21 and P53 genes in glioblastoma multiforme (GBM) utilizing a Wistar rat model (148). A total of thirty-five male rats were systematically allocated into seven distinct groups, which comprised healthy controls, cancer controls, training, nano-curcumin, and combined training–nano-curcumin cohorts. GBM cells were administered into the frontal cortex, subsequent to which the rats participated in a four-week regimen of treadmill training and/or were administered nano-curcumin (80 mg/kg/day). Gene expression analysis employing real-time PCR techniques indicated that miR-21 expression exhibited a significant reduction in the training, nano-curcumin, and combined groups in comparison to cancer controls. In contrast, P53 expression demonstrated a significant elevation in the nano-curcumin and combined groups. These findings indicated that endurance training and nano-curcumin may serve to impede tumor progression in GBM through the downregulation of the oncogenic miR-21 and the upregulation of the tumor suppressor P53, thereby underscoring a prospective non-pharmacological approach for the management of brain tumors (148) (**Table 3**).

**Table 3:** Molecular effects of exercise combined with polyphenols on cancer-related pathways: preclinical and clinical evidence.

Model/Participants	Intervention	Duration	Molecular targets	Results/Effects	Ref
35 male Wistar rats, 7 groups (5 rats each) with glioblastoma induced in frontal cortex	Endurance training (treadmill) and/or nano-curcumin (80 mg/kg orally)	4 weeks	miR-21, P53	miR-21 ↓, P53 ↑	(148)
40 female BALB/c mice with 4T1 breast cancer, 4 groups	Endurance training (40 min, 60–65% vVo2peak) and/or curcumin oral gavage	5 weeks	miR-126, Angiopoietin-1	miR-126 ↑, Angiopoietin-1 ↓	(149)
40 men with prostate cancer, 4 groups (10 each)	Aerobic exercise (60–90 min, 3x/week, 50–70% HRmax) and/or pomegranate juice (100 cc, 3x/week)	8 weeks	miR-21, miR-155, P53	miR-21 ↓, miR-155 ↓, P53 ↑	(150)
40 women recovering from breast cancer, 4 groups	Aerobic exercise (60–90 min, 3x/week, 50–70% HRmax) and/or pomegranate juice (100 cc before exercise)	8 weeks	miR-21, miR-155	miR-21 ↓, miR-155 ↓	(151)
24 male BALB/c mice with CT26-induced colorectal cancer, 4 groups + 6 healthy control	HIIT (75–90% max speed, 5–16 sets, 15° incline) and/or pomegranate juice (5% daily drinking water)	8 weeks	G-CSFR, JAK2, STAT3, MAPK, ERK	G-CSFR ↓, JAK2 ↓, STAT3 ↓, MAPK ↓, ERK ↓	(152)
40 male Wistar rats with glioblastoma, 5 groups	Aerobic exercise (treadmill, 18 m/min, 25–40 min, 3x/week) and/or nano-curcumin (80 mg/kg)	4 weeks	TGF-β1, TRAF6, CTGF	TGF-β1 ↓, TRAF6 ↓, CTGF ↓	(153)
24 female Balb/C mice with 4T1 breast cancer	Aerobic exercise ± nano-curcumin + doxorubicin	35 days (exercise 3x/week, nano-curcumin daily)	CASP3, CASP9, BAX, BCL2	CASP3 ↓, CASP9 ↓, BAX ↓, BCL2 ↑ (nano-curcumin), BCL2 → (exercise alone)	(154)
36 female Balb/C mice with 4T1 breast cancer	Aerobic exercise ± curcumin + doxorubicin	6 weeks (5x/week, 30 min)	CASP3, BCL2	CASP3 ↓, BCL2 ↑	(155)
40 male Copenhagen rats with prostate tumors	Aerobic exercise (ET) ± pomegranate juice (PJ)	4 weeks	Ki67, ERK, BCL2, antioxidant defenses, oxidative stress markers	Ki67 ↓ (ET or PJ), ERK ↓ (ET or PJ), BCL2 ↓ (PJ), antioxidant defenses ↑ (ET or PJ), combination ET+PJ → on BCL2 & antioxidant adaptation	(156)
BALB/c mice with 4T1 breast cancer	Regular exercise ± daidzein (145 mg/kg)	42 days (20 days pre-exercise, 22 days treatment)	NK cells, Fas/FasL mitochondrial apoptosis pathway	Tumor growth ↓ (exercise or daidzein), synergistic tumor inhibition ↑ (exercise+daidzein), NK cell activity ↑, apoptosis ↑ via Fas/FasL	(157)
Male Sprague-Dawley rats with azoxethane-induced colon cancer	Aerobic exercise ± aqueous extract of P. oleracea	8 weeks (5x/week, 60 min/session)	BAX, BCL2, BAX/BCL2 ratio, Caspase-3	BAX ↑, BAX/BCL2 ratio ↑, Caspase-3 ↑, BCL2 ↓	(158)

AE, Aerobic Exercise; ET, Exercise Training; HIIT, High-Intensity Interval Training; N-CUR, Nano-Curcumin; PJ, Pomegranate Juice; DOX, Doxorubicin; BAX, Bcl-2-associated X protein; BCL-2, B-cell lymphoma 2; CASP3, Caspase-3; CASP9, Caspase-9; TGF-β1, Transforming Growth Factor-beta 1; TRAF6, TNF Receptor Associated Factor 6; CTGF, Connective Tissue Growth Factor; NK, Natural Killer cells; miR, microRNA; ERK, Extracellular Signal-Regulated Kinase; G-CSF, Granulocyte Colony-Stimulating Factor; JAK2, Janus Kinase 2; MAPK, Mitogen-Activated Protein Kinase; P53, Tumor Protein p53.

Another investigation assessed the impact of endurance training in conjunction with curcumin on tumor development and the intratumoral expression levels of angiomiR-126 and Angiopoietin-1 in female BALB/c murine models afflicted with 4T1 breast cancer. A total of forty mice were systematically allocated into four distinct experimental groups: control, endurance training (E), curcumin (CC), and a combination of endurance training and curcumin (EC). The training regimen consisted of 40 minutes of exercise at an intensity of 60–65% vVO_2_peak, conducted five days per week over a duration of five weeks, while curcumin was administered via oral ingestion six days per week. Tumor specimens were harvested 24 hours subsequent to the final training session, and gene expression analysis was performed utilizing quantitative reverse transcription polymerase chain reaction (qRT-PCR). The findings indicated a significant reduction in tumor proliferation, an upregulation of miR-126, and a downregulation of Angiopoietin-1 across all intervention groups when compared to the control group. Notably, the combined EC group demonstrated the most marked effects (P<0.001) (149).

A quasi-experimental investigation assessed the effects of an eight-week regimen of aerobic exercise and pomegranate juice supplementation on microRNAs and the tumor suppressor protein P53 in a sample of 40 male individuals diagnosed with prostate cancer (mean age 61 years). Participants were allocated randomly into four distinct groups: control, exercise, supplementation, and a combined exercise–supplementation group. The exercise cohorts engaged in aerobic training sessions three times weekly for a duration of 60 to 90 minutes at an intensity corresponding to 50 to 70% of their maximum heart rate, whereas the supplementation cohorts ingested 100 mL of pomegranate juice three times per week. Blood samples were obtained 48 hours prior to and following the intervention. The findings indicated that the combined exercise–juice group experienced a statistically significant reduction in the levels of miR-21 and miR-155 (P = 0.001) along with a notable elevation in P53 protein levels (150).

Another semi-experimental investigation evaluated the impact of an eight-week regimen of aerobic exercise in conjunction with pomegranate juice on serum concentrations of oncogenic microRNAs among 40 female participants undergoing recovery from breast cancer, with a mean age of 42.45 years. The subjects were allocated randomly into four distinct groups: control, pomegranate juice, aerobic exercise, and a combination of aerobic exercise and pomegranate juice. The exercise sessions were conducted for a duration ranging from 60 to 90 minutes, three times weekly, maintaining an intensity of 50 to 70% of the target heart rate, while participants ingested 100 mL of pomegranate juice prior to each exercise session. Blood specimens were obtained 48 hours prior to and following the intervention, and the microRNAs miR-21 and miR-155 were quantified utilizing RT-PCR methodology. The findings indicated that the combined aerobic and pomegranate intervention led to a statistically significant reduction in serum levels of miR-21 and miR-155 when compared to the control group (P = 0.001) (151).

The aim of a study was to examine the implications of high-intensity interval training (HIIT) in conjunction with pomegranate juice (PJ) supplementation on the granulocyte-colony stimulating factor (G-CSF) signaling pathway within a murine model of colorectal cancer (CRC) induced by CT26 cells. A total of twenty-four male BALB/c mice diagnosed with CRC were systematically allocated into four experimental cohorts: CRC control, PJ, HIIT, and HIIT+PJ, in addition to six healthy control subjects. The HIIT regimen was administered over a period of eight weeks, comprising 5–16 high-intensity sets conducted at 75–90% of maximum velocity on a 15° incline, while PJ constituted 5% of the daily fluid intake. Induction of CRC markedly elevated the intestinal expression levels of G-CSFR, JAK2, STAT3, MAPK, and ERK. The intervention groups, most notably HIIT+PJ, exhibited statistically significant reductions in the expression of these genes compared to CRC control subjects (P ≤ 0.02), with synergistic effects that exceeded the outcomes of either intervention in isolation (152).

Overall, these investigations elucidated that the integration of physical exercise with polyphenols influences critical molecular pathways implicated in oncogenesis. Endurance or aerobic training, high-intensity interval training (HIIT), and supplementation with curcumin or pomegranate result in the downregulation of oncogenic microRNAs such as miR-21, miR-155, and angiomiR-126, while simultaneously promoting the upregulation of tumor suppressor proteins including P53 and Bax, thereby facilitating the process of apoptosis. Furthermore, these interventions impede pro-tumorigenic signaling cascades, comprising Angiopoietin-1, G-CSF/JAK2/STAT3/MAPK/ERK, and TGF-β–mediated fibrotic and epigenetic alterations, which contribute to the attenuation of tumor growth, angiogenesis, and inflammatory responses. The synergistic effects of these combination therapies typically surpass those of individual interventions in enhancing molecular modulation, thereby indicating that the synergistic relationship between polyphenols and exercise may represent a viable non-pharmacological approach for tumor suppression, mitigation of oxidative stress, and promotion of functional recovery across various cancer phenotypes.

Taken together, the evidence reviewed in this article suggests that the most distinctive contribution of polyphenol–exercise synergy in cancer lies not in short−term antioxidant effects but in its potential to reshape the epigenetic and fibrotic landscape that constrains long−term recovery. Across models, TGF−β emerges as the central node linking cytotoxic therapies, chronic inflammation, fibroblast activation and myofibroblast persistence, excessive collagen deposition, and the progressive stiffening that underlies contractures and movement limitation. Exercise alone can partially counter this by promoting mechano−dependent anabolic and anti−fibrotic signaling, but in the context of sustained TGF−β drive and epigenetic “locking” of fibrotic gene programs (via DNA methylation, histone marks, and non−coding RNAs), its effects are often incomplete and fragile. Polyphenols that target TGF−β–related oxidative and inflammatory signaling, redox−sensitive epigenetic modifiers, and apoptosis regulators (including the Bax/Bcl−2 axis) may therefore act as epigenetic co−factors: they do not replace the mechanotransductive stimulus of rehabilitation but can make tissues more receptive to it by loosening fibrotic transcriptional programs, supporting survival of non−malignant muscle and vascular cells, and dampening maladaptive apoptosis and senescence. At present, this model rests largely on preclinical and mechanistic evidence, with relatively sparse and heterogeneous human data in oncology and survivorship settings; accordingly, the reliability of direct clinical inferences is moderate at best. Nevertheless, the convergence of animal epigenetic data, TGF−β/fibrosis biology, and early human observations in other high−stress conditions supports a coherent, biologically plausible framework in which structured exercise provides the direction of adaptation and polyphenols refine the epigenetic and fibrotic “terrain” on which that adaptation unfolds. For cancer rehabilitation, this implies that carefully timed, disease−specific polyphenol–exercise interventions may, in principle, shift survivors from a trajectory of persistent fibrotic remodeling and reduced plasticity toward one of greater reversibility of tissue stiffness, improved muscle recoverability, and more durable functional gains, a hypothesis that now requires rigorous, targeted clinical testing in well−phenotyped oncologic cohorts.

## 8 Polyphenol–exercise synergy on modulating fibrotic pathways (e.g., TGF-β/SMAD)

An investigation analyzed the impact of sustained aerobic exercise and nano-curcumin supplementation on cardiac fibrosis via the TGF-β1/TRAF6/CTGF signaling pathway in rats afflicted with glioblastoma. A total of forty male Wistar rats were allocated into five distinct groups: a healthy control group, a tumor group, a tumor plus exercise (AE) group, a tumor plus nano-curcumin (N-CUR) group, and a tumor plus AE plus N-CUR group. Glioblastoma cells were introduced into the frontal cortex, and the intervention duration was established at four weeks. Aerobic exercise was conducted on a treadmill for durations ranging from 25 to 40 minutes, three times per week, while nano-curcumin was administered via oral route at a dosage of 80 mg/kg/day. The induction of tumors resulted in a significant elevation of TGF-β1, TRAF6, and CTGF mRNA levels within myocardial tissue. Both the AE and AE+N-CUR groups demonstrated a pronounced downregulation of TGF-β1 in comparison to tumor control subjects, with the combined treatment exhibiting the most substantial effect. These results imply that physical exercise, particularly when augmented with curcumin, offers protective effects against tumor-associated cardiac fibrosis by modulating the TGF-β1/TRAF6/CTGF signaling cascade (153).

## 9 Polyphenol–exercise synergy on modulating Bax/Bcl-2

A study assessed the implications of aerobic exercise and nano-curcumin micelles on the markers of cardiac apoptosis in BALB/C murine models afflicted with 4T1 breast cancer while receiving doxorubicin therapy. A total of twenty-four female mice were allocated into four distinct experimental groups: doxorubicin monotherapy, doxorubicin plus aerobic exercise, doxorubicin supplemented with nanocurcumin, and doxorubicin combined with both aerobic exercise and nanocurcumin. Aerobic exercise was conducted over a period of several weeks, while nanocurcumin was administered as a therapeutic adjunct. Analysis of gene expression demonstrated that aerobic exercise led to a significant attenuation of CASP3, CASP9, and Bax levels, whereas Bcl-2 expression remained stable. The administration of nanocurcumin resulted in a reduction of CASP3 and CASP9 levels, an elevation of Bcl-2, but did not influence Bax expression. The concomitant application of both treatments yielded the most significant protective effect (154) (**Table 3**).

The purpose of a study was to determine the impact of aerobic exercise in conjunction with curcumin on apoptotic indicators within the hepatic tissues of Balb/c murine models afflicted with 4T1 breast carcinoma during the administration of doxorubicin. A total of thirty-six female mice were systematically allocated into six distinct cohorts: healthy control, cancer control, cancer+doxorubicin, cancer+doxorubicin+exercise, cancer+doxorubicin+curcumin, and cancer+doxorubicin+exercise+curcumin. The aerobic exercise regimen entailed thirty-minute treadmill sessions conducted five days per week over a six-week duration, whereas curcumin was delivered via oral administration. Gene expression analysis demonstrated that the synergistic application of exercise and curcumin resulted in a significant reduction of Caspase-3 levels and an enhancement of Bcl-2 expression in comparison to the cancer control group. The amalgamated intervention (exercise+curcumin+doxorubicin) manifested the most pronounced anti-apoptotic efficacy (155).

A preclinical investigation examined the ramifications of exercise training (ET), pomegranate juice (PJ), and their synergistic application on the progression of prostate cancer in male Copenhagen rats. A total of forty rats diagnosed with prostate tumors were allocated into four distinct groups: control, PJ, ET, and PJ+ET. Administration of PJ (750 µL/day) and ET (treadmill, 5 days/week) independently resulted in a reduction of tumor proliferation (Ki67), a decrease in ERK phosphorylation, a lower expression of Bcl-2, and an enhancement of antioxidant defenses in both muscle and plasma, concomitantly diminishing oxidative stress markers. Nevertheless, the combined treatment of PJ+ET unexpectedly negated these effects, failing to impede tumor growth or bolster antioxidant defenses (156).

An investigation demonstrated the synergistic interactions of habitual physical activity and daidzein supplementation in relation to breast cancer pathogenesis in BALB/c mice harboring 4T1 tumors. The subjects were subjected to a regimen of treadmill exercise over a span of 20 days (15 m/min, 60 min/day), which was subsequently followed by 22 days of daidzein administration at a dosage of 145 mg/kg. Each intervention, when assessed in isolation, demonstrated an inhibitory effect on tumor proliferation; however, the combined approach revealed a synergistic attenuation of tumor advancement (P<0.01). At a mechanistic level, the co-administration of exercise and daidzein facilitated the mobilization and redistribution of natural killer (NK) cells, mediated by elevated levels of epinephrine and IL-6, thereby augmenting immune surveillance. Furthermore, the synergistic treatment activated the Fas/FasL-mediated mitochondrial apoptosis pathway, thereby promoting the apoptosis of cancerous cells (157).

The aim of a research was to examine the impact of aerobic exercise and the aqueous extract of Portulaca oleracea on the expression of apoptotic genes in male Sprague-Dawley rats diagnosed with azoxymethane-induced colon carcinoma. A total of thirty rats were systematically allocated into five distinct groups: healthy control, tumor control, tumor with exercise, tumor with Portulaca extract, and tumor with both exercise and extract. The exercise regimen entailed treadmill sessions lasting 60 minutes, conducted five days per week over a duration of eight weeks, while the extract was administered via the intraperitoneal route at a dosage of 75 mg. The findings revealed that both individual and combined interventions led to a notable increase in Bax, Caspase-3, and the Bax/Bcl-2 ratio, concurrently resulting in a reduction of Bcl-2 expression (p<0.001). The combined therapeutic approach exhibited effects akin to those of the individual treatments, thereby suggesting a synergistic modulation of apoptotic pathways (158).

These investigations collectively elucidate that physical exercise, in conjunction with polyphenols or bioactive phytochemical extracts, influences critical apoptotic and immune pathways across a spectrum of malignancies. Interventions such as aerobic exercise or high-intensity interval training (HIIT) combined with curcumin, daidzein, pomegranate juice, or Portulaca oleracea result in the upregulation of pro-apoptotic factors, which include Bax, Caspase-3, Caspase-9, and Fas/FasL, concomitantly leading to the downregulation of the anti-apoptotic protein Bcl-2. Moreover, these interventions modulate the TGF-β1/TRAF6/CTGF signaling cascade, mitigate oxidative stress, and augment natural killer (NK) cell mobilization through the action of IL-6 and epinephrine. Collectively, these molecular alterations facilitate apoptosis, impede tumor proliferation, and safeguard non-target tissues during chemotherapy. The synergistic interactions between exercise and bioactive compounds typically yield more pronounced effects than those observed with isolated interventions, thereby indicating a promising non-pharmacological approach for cancer management and the preservation of tissue integrity.

Across the studies considered in this section, a consistent theme is that polyphenol–exercise combinations tend to re−balance the Bax/Bcl−2 axis away from indiscriminate cell loss in normal tissues and toward more selective, context−appropriate apoptosis, although the strength of this conclusion varies by model and outcome. In tumor−bearing or therapy−exposed settings, exercise alone often shows modest, directionally favorable shifts (e.g., increased Bax and/or decreased Bcl−2 in malignant tissue, or preservation of Bax/Bcl−2 homeostasis in skeletal muscle), but these effects can be attenuated by sustained oxidative stress and pro−fibrotic cytokine drive. When polyphenols are layered onto exercise—particularly those with documented actions on mitochondrial integrity, NF−κB/Nrf2 signaling, and upstream regulators of intrinsic apoptosis—the Bax/Bcl−2 changes are generally larger and more coherent, with several preclinical models demonstrating concurrent improvements in mitochondrial function, reduced cytochrome c release, and attenuated caspase activation in non−tumor muscle, while maintaining or even enhancing pro−apoptotic signaling in tumor tissue. However, the direction of modulation is not uniform: in some models the combined intervention predominantly raises Bax and lowers Bcl−2 in aberrant or fibrotic tissue, whereas in others it mainly protects healthy myocytes by stabilizing Bcl−2 and preventing excessive Bax−driven apoptosis during or after intense loading. This pattern underscores that Bax/Bcl−2 is not a simple “good vs. bad” switch but a context−dependent node that can be steered in opposite directions depending on the cellular target and disease stage. Overall, the preclinical evidence for a biologically plausible, mechanistically anchored synergy between polyphenols and exercise on Bax/Bcl−2 regulation is moderately strong within individual models, but direct clinical evidence in cancer survivors remains sparse and heterogeneous. Consequently, while Bax/Bcl−2 offers an attractive mechanistic lens to understand how combined nutritional–mechanical strategies might simultaneously protect muscle, modulate tumor behaviour, and mitigate therapy−related tissue damage, the translational reliability of these findings should currently be regarded as moderate, and they should primarily guide the design of future hypothesis−driven human trials rather than immediate clinical practice.

## 10 Limitations and future directions

Despite the growing body of literature examining interactions between polyphenol supplementation and exercise, evidence specifically addressing their combined effects within oncology populations and tumor-associated mechanobiological dysfunction remains limited and heterogeneous. These limitations do not invalidate the mechanistic convergence discussed in this review, but they define the translational boundaries within which current conclusions can be cautiously interpreted.

A central limitation lies in the pronounced heterogeneity of both clinical cancer populations and intervention designs. Cancer type, stage, treatment modality (chemotherapy, radiotherapy, surgery, immunotherapy), and cachexia status profoundly influence molecular signaling within the tumor–muscle–matrix axis. Exercise protocols vary substantially in modality, intensity, and timing relative to active treatment, while polyphenol interventions differ in compound class, bioavailability, dosing strategy, and duration. This variability complicates cross-study comparison and restricts the ability to establish unified mechanistic models or clinically actionable recommendations.

Consequently, observed effects likely reflect context-specific modulation of oncogenic, inflammatory, and fibrotic signaling pathways rather than universally transferable responses. Closely related to this issue is inconsistency in outcome prioritization. Many studies emphasize circulating cytokines, oxidative stress indices, or isolated signaling molecules as primary endpoints. While these biomarkers provide mechanistic insight, they do not consistently correlate with clinically meaningful outcomes such as muscle strength preservation, mitigation of fibrosis, reduction of biomechanical stiffness, or rehabilitation responsiveness in cancer survivors. The frequent dissociation between molecular markers and functional recovery underscores the need for integrated frameworks linking epigenetic regulation, extracellular matrix remodeling, and measurable biomechanical performance.

Another critical limitation concerns the imbalance between preclinical oncology models and human evidence. Animal and *in vitro* studies robustly demonstrate that polyphenols modulate epigenetic regulators (e.g., microRNAs, HDAC activity, DNA methylation) and fibrotic pathways such as TGF-β/SMAD, yet these mechanisms have not been systematically validated in well-controlled human oncology trials that concurrently assess molecular, structural, and functional endpoints. Differences in tumor burden, metabolism, treatment exposure, and dosing strategies constrain direct translation. As a result, many mechanistic interpretations in cancer patients remain inferential rather than directly demonstrated.

Population representation further limits generalizability. Most exercise–nutrition trials have been conducted in healthy or athletic populations, whereas data in cancer patients particularly those with treatment-induced fibrosis, cachexia, or neuromuscular impairment, remain comparatively sparse. This gap is especially relevant given that altered inflammatory tone, mitochondrial dysfunction, and epigenetic instability in oncology settings may fundamentally modify responsiveness to both exercise and polyphenol interventions. Temporal scope represents an additional constraint. Many investigations focus on short-term recovery windows or brief intervention periods. The long-term implications of sustained polyphenol supplementation during prolonged cancer treatment, survivorship rehabilitation, or chronic fibrotic remodeling remain insufficiently characterized. It is unclear whether chronic modulation of epigenetic and inflammatory pathways enhances adaptive resilience or potentially interferes with necessary stress-signaling processes during tumor therapy.

Taken together, these considerations highlight the importance of interpreting current findings within a nuanced, oncology-specific framework. Rather than supporting prescriptive conclusions, existing evidence underscores the need for mechanistically integrated, biomarker-guided approaches tailored to cancer stage, treatment exposure, and functional status. Future research should prioritize multimodal translational designs that simultaneously evaluate epigenetic biomarkers, tumor-derived signaling mediators, extracellular matrix remodeling indices, and advanced biomechanical assessments in cancer populations. Greater methodological alignment in exercise prescription, polyphenol dosing, and molecular outcome selection would substantially enhance interpretability. In particular, strategically matching supplementation timing with treatment phase (e.g., peri-chemotherapy vs survivorship rehabilitation) may clarify whether polyphenols function primarily as modulators of treatment-induced toxicity, facilitators of fibrotic attenuation, or enhancers of regenerative adaptation.

Future investigations should also emphasize clinically meaningful endpoints. Integrating mechanobiological metrics such as tissue stiffness, muscle architecture, strength recovery, and activity variability, with molecular signatures of epigenetic and fibrotic regulation would provide a more comprehensive understanding of functional restoration in oncology settings. Expanding research into underrepresented cancer subgroups including older adults, patients with cachexia, and individuals experiencing radiotherapy-induced fibrosis, remains a priority. Longitudinal studies are particularly needed to determine whether combined polyphenol–exercise strategies can meaningfully support rehabilitation adherence, functional independence, and long-term survivorship outcomes. From a mechanistic standpoint, bridging preclinical epigenetic insights with human tumor–muscle interactions are essential. Advances in transcriptomics, proteomics, metabolomics, and circulating exosomal profiling provide promising tools to validate whether pathways such as TGF-β/SMAD signaling, microRNA regulation, and HDAC modulation are coordinately reprogrammed in response to combined interventions. Ultimately, the field must move beyond asking whether polyphenols exert general ergogenic effects and instead clarify how targeted molecular–mechanobiological reprogramming of the tumor–muscle–matrix axis may reduce biomechanical dysfunction, attenuate injury risk during rehabilitation, and optimize functional recovery in cancer patients. Such a shift toward precision, systems-level investigation will better align future research with the clinical realities of oncology care.

## 11 Conclusion

This review advances a coherent perspective in which functional activity capacity, rehabilitation outcomes, and biomechanical integrity in cancer patients are shaped by a joint nutritional–mechanical influence, rather than by exercise or supplementation in isolation. Across the evidence base, exercise consistently acts as the dominant driver of adaptation, while selected polyphenols appear to modulate the molecular and epigenetic “terrain” of the tumor–muscle–matrix axis. Evidence from both preclinical cancer models and human oncology studies suggests that polyphenols can shift recovery trajectories by modulating epigenetic regulators (e.g., microRNAs, HDACs) and fibrotic pathways (e.g., TGF-β/SMAD), enhancing neuromuscular resilience, ECM compliance, and activity quality.

Compound-specific effects remain context-dependent. Curcumin, the most extensively studied polyphenol, demonstrates selective benefits depending on supplementation timing and outcome prioritization. Resveratrol may preferentially support preservation of high-intensity muscular output, whereas quercetin may enhance anabolic signaling and tissue resilience rather than producing uniform antioxidant effects. Whole-food polyphenols, such as those in blueberries or tea, further illustrate that functional recovery and perceptual outcomes (e.g., pain, soreness) can diverge, highlighting the importance of integrative mechanobiological and molecular assessment.

Supporting mechanistic evidence from preclinical oncology and rehabilitation models provides biological plausibility across tissues: muscle and connective tissue studies converge on modulation of oxidative stress, inflammation, mitochondrial function, protein turnover, and fibrotic remodeling; neurorehabilitation models suggest amplification of exercise-driven plasticity through BDNF/TrkB, SIRT1, and related pathways; organ-level models indicate potential cardioprotective and hepatoprotective effects, although additive benefits depend on the distinct molecular pathways targeted by each intervention.

Taken together, the available human and preclinical data suggest that polyphenol–exercise synergy is a biologically plausible and clinically promising strategy to modulate tumor–muscle–matrix crosstalk, but the evidence base is not yet strong enough to support precise, universally applicable prescriptions. Across human trials in EIMD and at−risk clinical populations, polyphenol doses typically fall within the low−to−moderate pharmacological range (e.g., curcumin ≈ 150–1000 mg/day or equivalent, quercetin ≈ 500–1000 mg/day, mixed berry/blueberry polyphenols in amounts achievable by diet), administered for days to a few months, while exercise interventions are predominantly structured, supervised protocols involving eccentric or resistance exercise, high−intensity interval or anaerobic sessions, and/or moderate−to−vigorous endurance training performed 2–5 times per week. Within this envelope, the most consistent benefits appear when polyphenols are coordinated with repeated exercise exposure over weeks, rather than as a single acute dose, and when exercise provides a clearly progressive mechanical stimulus sufficient to drive adaptation. However, study heterogeneity in formulations, timing (pre− vs post−exercise), and adherence, combined with relatively small sample sizes and underpowered designs, means that even when group differences reach conventional statistical significance, the overall statistical reliability is best described as moderate: effect sizes are often modest, confidence intervals wide, and replication limited. Preclinical models provide stronger internal validity, showing convergent improvements in oxidative–inflammatory balance, TGF−β−driven fibrosis, neuromuscular plasticity, and Bax/Bcl−2−mediated apoptotic control when polyphenols are paired with structured training or rehabilitation, but translational certainty to human oncology remains moderate due to species, dosing, and disease−model differences. Consequently, current evidence supports viewing polyphenols as adjunctive, context−sensitive modulators to be integrated into individualized, disease− and treatment−specific exercise programs, rather than as stand−alone agents or as a basis for rigid dosing algorithms. Future trials should prioritize larger, rigorously controlled, mechanistically phenotyped studies that systematically vary polyphenol dose, formulation, timing, and exercise modality/duration to determine whether the promising effects on recovery, function, epigenetic remodeling, and Bax/Bcl−2 balance can be translated into statistically robust and clinically meaningful gains for cancer patients and survivors.

Overall, the current body of evidence suggests that polyphenols and related phytochemicals may influence skeletal muscle adaptation, injury responses, and recovery through mechanisms involving oxidative stress regulation, inflammation control, mitochondrial function, and cell-death signaling. However, the strength of the evidence varies considerably across study types, with mechanistic support largely derived from controlled preclinical models and more heterogeneous findings emerging from human studies. Importantly, the biological effects of these compounds appear to be context-dependent, influenced by factors such as dosage, duration of supplementation, exercise modality, and physiological or disease state. Taken together, the key take-home message is that while polyphenol-based interventions show promising potential as modulators of muscle health and rehabilitation, particularly in conditions involving oxidative or inflammatory stress, the current evidence base supports cautious interpretation and highlights the need for well-designed, standardized clinical trials to clarify optimal dosing strategies, exercise contexts, and translational relevance.
